# Discovery of Novel Human Gene Regulatory Modules from Gene Co-expression and Promoter Motif Analysis

**DOI:** 10.1038/s41598-017-05705-2

**Published:** 2017-07-17

**Authors:** Shisong Ma, Michael Snyder, Savithramma P. Dinesh-Kumar

**Affiliations:** 1 School of Life Sciences, University of Science and Technology of China, Hefei, Anhui 230027 China; 2 Department of Plant Biology and the Genome Center, College of Biological Sciences, University of California, Davis, CA 95616 USA; 3 Department of Genetics, Stanford University, Stanford, CA 94305 USA

## Abstract

Deciphering gene regulatory networks requires identification of gene expression modules. We describe a novel bottom-up approach to identify gene modules regulated by *cis*-regulatory motifs from a human gene co-expression network. Target genes of a *cis*-regulatory motif were identified from the network *via* the motif’s enrichment or biased distribution towards transcription start sites in the promoters of co-expressed genes. A gene sub-network containing the target genes was extracted and used to derive gene modules. The analysis revealed known and novel gene modules regulated by the NF-Y motif. The binding of NF-Y proteins to these modules’ gene promoters were verified using ENCODE ChIP-Seq data. The analyses also identified 8,048 Sp1 motif target genes, interestingly many of which were not detected by ENCODE ChIP-Seq. These target genes assemble into house-keeping, tissues-specific developmental, and immune response modules. Integration of Sp1 modules with genomic and epigenomic data indicates epigenetic control of Sp1 targets’ expression in a cell/tissue specific manner. Finally, known and novel target genes and modules regulated by the YY1, RFX1, IRF1, and 34 other motifs were also identified. The study described here provides a valuable resource to understand transcriptional regulation of various human developmental, disease, or immunity pathways.

## Introduction

A gene regulatory network (GRN) describes how gene expression dynamics is regulated in an organism under different biological conditions. Building a GRN requires information concerning three domains - the components and circuits of the network, how these components and circuits are used under various conditions, and the output of the network, i.e. the dynamics of gene expression pattern. Over the past decade, enormous progress has been made in the first and third domains, but only minimal progress has been made to integrate these two domains that would enhance our knowledge with respect to the second domain.

Transcription factors (TF) bind to *cis*-regulatory sequences or motifs within a gene’s promoter and regulate expression. The binding of TFs to promoters of TF and non-TF genes constitutes the backbone of a GRN. Many TFs’ binding motifs have been characterized and listed in databases such as JASPAR and TRANSFEC^1, 2^. Recently, the human ENCODE project has mapped TF binding sites at the genome level using chromatin immunoprecipitation followed by high throughput sequencing (ChIP-Seq) and expression regulatory regions using DNase I hypersensitive sites sequencing (DNase-Seq)^3–5^. Data generated from these analyses have been used to derive the circuits and architectures of TF regulatory network^3–5^. Such studies lead to an extensive characterization of the components of regulatory network. Interactions between and combinations of these components provide a vast regulatory space and potential for gene expression regulation.

Human gene expression in various tissues, during development, or under diverse environmental conditions has also been cataloged systematically in NCBI GEO or ArrayExpress databases. These large datasets have been used to generate gene co-expression networks, in which genes with similar expression patterns were connected^6^. These networks effectively group genes with similar functions or functioning in the same processes, and have been used to analyze the transcriptome of the human brain, primary cell lines, and various tissues. This advanced the identification of, for example, specific molecular pathways in autism and amyotrophic lateral sclerosis^7–11^. Although these analyses catalogued distinct expression patterns, they failed to predict a specific TF or group of TFs that regulate the identified co-expressed genes in the network.

A substantial amount of data on the components and the output of human GRNs have now been accumulated. However, very limited efforts have been made to integrate these datasets. Although an enormous amount of TF-binding site data is available in public repositories, it is difficult to uncover the relevant components for specific conditions without further careful informatics-based analyses. In addition, the co-expressed gene groups derived from co-expression networks provide little insights into the regulatory TF which drive the expression of genes in the co-expression network.

To overcome these shortcomings, we recently described novel methods to integrate the two distinct components of a regulatory network for a plant model system, *Arabidopsis thaliana* ^12^. We conducted promoter motif analysis overlying the gene co-expression network and identified target genes regulated by specific *cis*-regulatory motifs *via* motif enrichment and motif position bias towards transcription starting site (TSS). The target genes were then used to identify motif-regulated gene co-expression modules. The relevant TFs driving the expression of genes within the network were then identified. Comparing to other co-expression network studies, our approach provided the much-needed mechanistic insights on how gene co-expression networks are regulated by different TFs^12^.

Here, we describe a human GRN by merging both regulatory components and gene co-expression networks. We used data from 948 microarray datasets from ArrayExpress^13^ to build a human gene co-expression network. Promoter motif analysis over the network identified many target genes and co-expression modules *via* motif enrichment and motif position bias methods. Many known and novel modules regulated by the nuclear factor Y (NF-Y), specificity protein 1 (Sp1), and 37 other *cis*-regulatory motifs were identified. The interaction between NF-Y and Sp1 TFs and their target genes were validated using ENCODE ChIP-seq data. Interestingly, while modules regulated by NF-Y are mainly involved in house-keeping functions, the Sp1 motif targets include both house-keeping and tissue specific gene expression modules. The derived Sp1 modules were superimposed on various genomic and epigenomic data to provide insights into how Sp1 regulates diverse gene targets. Modules were also identified for 37 additional motifs, such as the YY1, RFX2, and IRF1 binding motifs. Our approach identified numerous novel target genes for various motifs, and organized these targets into co-expression modules. The modules then enabled integrating various genomic/epigenomic data into a coherent regulatory system, providing a valuable resource to identify transcriptional regulators for various human developmental, disease, or immunity pathways.

## Results

### Human gene co-expression network

**Figure 1:**
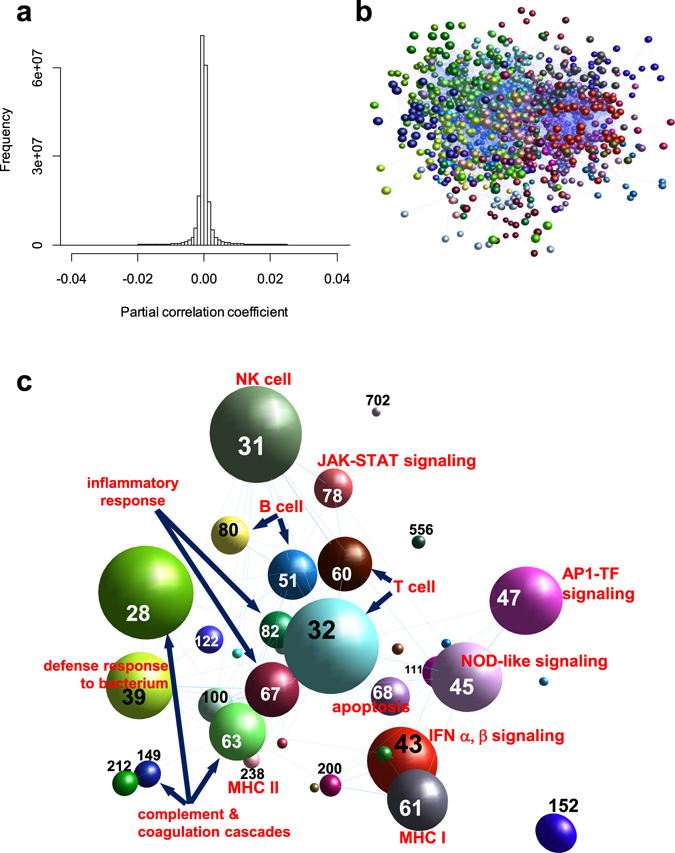
Characterization of the human co-expression network. (**a**) Histogram showing the distribution of the *partial correlation coefficient* (*pcor*) between gene pairs. Most gene pairs show *pcors* between -0.02 and 0.02. (**b**) A sub-network for immunity-related modules extracted from the entire gene co-expression network. In the network, each sphere represents a gene, and connection between genes indicates their similar expression pattern. Genes are colored according to their module identities. (**c**) A simplified version of the sub-network from B is shown. The genes from the same module are represented by a single sphere. The size of the sphere is proportional to the number of genes within a module. The number shown within the module sphere represents module # shown in Supplementary Dataset 1. The network is shown in a 3-D space layout and some modules (e.g. #477 and #851) are hidden behind modules in foreground.

We constructed a gene co-expression network for 19,718 human genes based on the graphical Gaussian model (GGM)^14, 15^ using Affymetrix U133 Plus 2.0 microarray data deposited in the ArrayExpress database^13^. GGM uses partial correlation coefficient (*pcor*), the correlation between two genes after removing the effects from other genes, to measure gene expression similarity. *Pcor* performs better than the conventional Pearson’s correlation coefficient in gene network analyses^15, 16^. As shown in Fig. 1a, 97% of the gene pairs have their *pcor* values in the range of −0.01 to 0.01, indicating no correlation. The gene pairs with *pcor* >= 0.04 (false discover rate, FDR, 3.56E-15) were selected. As a result, 186,132 significantly correlated gene pairs (0.095% of all possible pairs) among 19,376 genes were used to construct a human GGM gene co-expression network.

The derived network consolidated into 930 clusters *via* the Markov Cluster Algorithm (MCL) (Supplementary Dataset 1)^17^. These clusters were treated as co-expression modules. Gene ontology (GO) analysis identified 36 modules enriched with genes functioning in immunity pathways (pValue < 1E-5) (Supplementary Dataset 2). A sub-network extracted for these 36 modules (Fig. 1b and c) includes multiple aspects of immune signaling pathways such as B-cells (module #51, 80, 477), T-cells (#32, 60, 851), and nature killer cells signaling (#31, 556, 702), p53 signaling and apoptosis (#68), Interferon α/β signaling (#43, 199), MHC I (#61) and MHC II (#238) antibody processing and presentation, complement & coagulation cascades (#28, 63, 149), NOD NLR singling (#45), and inflammatory response (#67, 82). In addition to immune signaling modules, our network identified another 142 modules enriched with genes functioning in development, metabolism, or house-keeping functions and other signaling pathways (Supplementary Dataset 1).

### Identification of targets of promoter motifs from gene co-expression network

**Figure 2:**
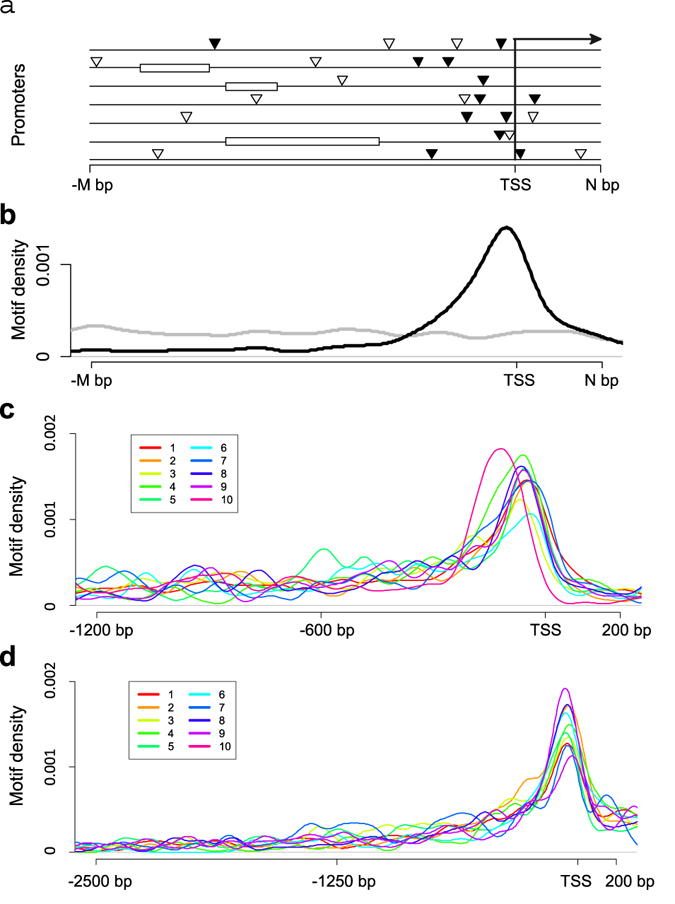
Motif target identified through motif position bias towards TSS. (**a**) A diagram showing a group of promoters. Within the promoters, a random/non-functional motif (grey triangle) distributes randomly along the promoters, while a functional motif (solid black triangle) distributes towards the transcription start site (TSS). The white bars represent transposon or repetitive sequence in the promoters, which are excluded from motif analysis. The arrow indicates the direction of transcription. (**b**) A representative distribution of a random/non-functional motif (grey line) and a functional motif (black line) with bias distribution along promoters. (**c**) Distribution of the NF-Y (CCAAT) motif within gene promoters from the modules regulated by this motif. Data from the first 10 modules shown in Supplementary Dataset 3 are indicated by different colors, as specified by the color key on the top left. (**d**) Distribution of the Sp1 motif within the gene promoters for modules regulated by the Sp1 motif. Data from first 10 modules shown in Supplementary Dataset 5 are indicated by different colors, as specified by the color key on the top left.

The gene co-expression network contains gene co-expression modules regulated by specific promoter motifs(s). A bottom-up approach was employed to identify such motifs-regulated modules. The target genes for a specific motif are identified by motif analysis^12^ over gene co-expression network. The target gene list is then used to detect if they form any modules. For each gene, the gene itself and its neighbor are treated as a group, and the gene itself as a seeded gene. The group’ promoters are then analyzed to see if the motif has enrichment within them (measured with a pValue *via* hypergeometric distribution), or if the motif has position bias distribution towards transcription start site (TSS) (measured with a Z score, see below for details). If the seeded gene’s promoter contains the motif, and the motif is enriched in the group’s promoters or demonstrates significant position bias towards TSS, all the genes within the group which contain the motif will be considered to be regulated by that motif (Fig. 2). A sub-network is then extracted for the target genes and used for gene co-expression modules detection.

While motif enrichment analysis has been widely used^18–20^, there has yet to be an efficient and accurate model to measure motif position bias, although such bias has been used in numerous reports as evidence of *bona*-*fide* motifs^21, 22^. We recently described a model based on discrete uniform distribution to measure such motif position bias^12^. Here, we expanded the model to accommodate more complex conditions. From the motif analyses, we first excluded sequences that form simple repeats or transposons within all promoters. A second consideration was based on that *cis*-regulatory motifs can be present either upstream or downstream of TSS (Fig. 2a). If an irrelevant motif were to have arisen randomly within a group of promoters, it will distribute uniformly along the promoters (Fig. 2b). The motif’s expected distance *E*(*d*) from TSS and its variance *V*(*d*) can be calculated (see Material & Methods for details). In contrast, many functional motifs distribute in a biased manner towards TSS with much smaller distance (Fig. 2b). For a given motif that appears *n* times within the same group of promoters, with mean distance of $\overline{|x|}$ from TSS, a Z score can be calculated *via* the following formula as a measurement of biased distribution.

1 $$Z=(E(d)-\overline{|x|})/\sqrt{V(d)/n}$$

The higher the Z score, the higher the chance that the motif is biasedly distributed towards TSS and the higher possibility that the genes are regulated *via* that motif. Using this approach, we identified many known and hitherto unknown target genes for NF-Y and Sp1 binding *cis*-regulatory motifs (Fig. 2c and d).

### Gene expression modules regulated by the NF-Y motif

The gene co-expression network was analyzed to identify potential targets regulated by the ubiquitously expressed NF-Y TFs that bind to the CCAAT motif^23^. This motif appears in the promoters of 11,998 human genes (61% of all genes analyzed). The motif enrichment method identified only 85 genes as NF-Y motif target genes with a pValue cutoff set at 1E-05. In contrast, the motif position bias analysis identified 3,062 genes as NF-Y motif target genes with Z scores >  = 3.5. These 3,062 genes include the 85 genes identified *via* motif enrichment method. The promoter sequences of all genes used in the network analysis were then randomized and subjected to the same analysis. In average 34 genes were identified as NF-Y motif targets in each permutation experiment. Thus the false discovery rate (FDR) for NF-Y motif target genes analysis is 1.1% (34/3062).

After identifying 3,062 NF-Y target genes by performing motif analysis over the whole co-expression network, we then asked if these target genes form any gene module or not. A sub-network was extracted from the whole co-expression network for these 3,062 target genes and clustered into 129 modules, 33 of which contain enriched GO terms (pValue < 5E-7, Table 1 and Supplementary Dataset 3). These modules are potentially regulated by the NF-Y TFs through the CCAAT motif. The CCAAT motif displays position bias towards the TSS among the genes within these modules (Fig. 2c). Many of the modules function in known pathways regulated by NF-Y TFs, such as the cell cycle (module #1), RNA/mRNA processing (#13, 16, 77), protein folding and ER related functions (#4, 26), cholesterol and lipid metabolism (#7, 48, 63), developmental patterning (#62, 68, 83), glucose and carboxylic acid metabolism (#11, 20), fatty acid oxidation (#49), and antigen processing via MHC class II (#29) (Table 1)^24–26^. Other modules indicate novel functions for human NF-Y, such as, Golgi vesicle transport (#18), protein polymerization (#22), circadian rhythmic regulation (#24), cilium organization and spermatogenesis (#36), gland development (#53), platelet activation (#59), and cellular response to lipopolysaccharide (#38) (Table 1). Supporting our findings for novel modules #36 and #53, a NF-YB homolog in *Schimidtea mediterranea* is required for male germ cell development, while NF-Y binding sites are required for basal transcription of *TBX3*, a key developmental regulator in module #53^27, 28^.

**Table 1:** NF-Y motif (CCAAT)-regulated modules.

Module No.	# of genes in the Module	GO Enrichment for the genes within the module			Binding of NF-YA/YB protein to the gene promoters within the module according to ENCODE data		
		Enriched GO	GO Enrichment pValue	Selected Genes in the module with the enriched GO term	# of Genes in the module with NF-YA/B bound CCAAT motif in promoter	Binding Enrichment, Fold change, compared to genome-wide average level	Binding Enrichment pValue
1	180	cell cycle	3.44E-76	BRCA1/BRCA2/MSH2	113	2.23	2.1455E-22
2	109	translational elongation	6.24E-37	RPSA/RPS19/RPL5	58	1.89	2.86376E-08
4	79	response to endoplasmic reticulum stress	2.86E-20	HSPA5/XBP1/HSP90B1	63	2.83	1.79692E-21
7	66	cholesterol biosynthetic process	1.62E-35	APOE/HMGCR/DHCR7	41	2.21	8.53E-09
8	64	cellular amino acid metabolic process	1.09E-10	ATF4/CTH/WARS	33	1.83	6.28382E-05
10	58	nucleosome organization	1.52E-31	HIST2H2BE/HIST1H1C/HIST1H1B	44	2.69	5.81052E-14
11	56	pyruvate metabolic process	8.06E-11	GAPDH/ENO2/LDHA	23	1.46	0.025308353
13	53	RNA splicing	7.55E-10	PPP2CA/DDX5/SRSF2	30	2.01	1.26722E-05
15	51	regulation of transcription, DNA-templated	5.22E-10	MBD2/MBD3/ZNF266	34	2.37	1.17185E-08
16	51	RNA processing	1.68E-10	SSB/NOLC1/TGS1	22	1.53	0.015358532
18	45	Golgi vesicle transport	1.16E-09	RP2/CREB3L2/USO1	21	1.66	0.006178619
19	44	regulation of transcription, DNA-templated	4.51E-21	IRAK4/ZNF267/ZNF92	30	2.42	3.91083E-08
20	38	organic acid catabolic process	1.76E-14	PPARA/GCDH/BCKDHB	24	2.24	7.13037E-06
22	35	protein polymerization	7.88E-12	TUBB3/TUBB/TUBA1A	10	1.01	0.542074745
24	35	circadian regulation of gene expression	3.78E-08	PER2/PER3/PER1	19	1.93	0.001000469
26	34	protein folding	7.22E-12	HSPD1/HSPA8/HSPA9	14	1.46	0.070324677
29	30	antigen processing and presentation of exogenous peptide antigen via MHC class II	7.47E-11	HLA-DRB1/HLA-DPB1/HLA-DRA	15	1.78	0.009139083
31	28	regulation of developmental process	1.54E-07	TP53/CDKN1B/JUN	16	2.03	0.001203638
36	25	cilium organization	3.45E-07	ODF2/TMEM231	7	0.99	0.582218333
38	23	cellular response to lipopolysaccharide	9.79E-08	IL1B/IL8/NFKBIA	8	1.24	0.308349607
42	22	cellular response to DNA damage stimulus	2.14E-10	CDKN1A/MDM2/BAX	14	2.26	0.000551652
44	22	regulation of transcription, DNA-templated	2.57E-11	ZNF350/ZNF667/ZNF569	8	1.29	0.261090348
45	22	cellular respiration	3.01E-08	ATP5B/PDHA1/NDUFS2	12	1.94	0.008155563
48	21	triglyceride metabolic process	1.40E-11	LIPE/DGAT2/GPD1	4	0.68	0.882989411
49	21	fatty acid oxidation	4.40E-08	CPT2/PDK4/ACADVL	11	1.86	0.016309781
53	18	gland development	1.29E-12	SHH/BMP4/SNAI2	8	1.58	0.103963857
59	16	platelet activation	1.56E-09	ILK/FLNA/ACTB	8	1.78	0.052838504
62	15	anterior/posterior pattern specification	1.89E-12	DKK1/HOXA10/HOXA1	3	0.71	0.838818923
63	14	regulation of plasma lipoprotein particle levels	3.62E-10	ABCA1/ABCG1/MYLIP	7	1.78	0.068971412
68	14	embryonic limb morphogenesis	1.06E-08	HOXD13/FGF9/HOXA13	1	0.25	0.990266126
77	12	mRNA processing	1.38E-07	HNRNPA1/HNRNPA2B1/PABPN1	3	0.89	0.700561294
83	10	anterior/posterior pattern specification	7.01E-09	HOXC13/HOXC6/HOXC9	7	2.49	0.007218374
85	10	regulation of cellular amino acid metabolic process	2.10E-13	PSMC5/PSMD2/PSMD1	2	0.71	0.819878552

We validated the binding of NF-Y TFs to the gene promoters within the above-described modules using ENCODE ChIP-Seq data^4, 25^ (Table 1). Among all the 11,998 genes with NF-Y motifs in their promoters used in our network analysis, 3,378 (or 28%) contained NF-Y motif site(s) that were bound by NF-YA and/or NF-YB protein in at least one of three human cell lines (K562, GM12878, and HeLa S3) used in the ENCODE ChIP-Seq analyses. Among the 3,062 NF-Y motif target genes identified from our network analyses, 1,508 (or 49%) contained NF-Y protein-bound NF-Y motif sites, representing a 1.75 (49%/28%) fold enrichment compared to the genome-wide level (pValue = 3.6E-187). Furthermore, 20 of the 34 NF-Y motif regulated modules that we identified in our analyses, including the ones with novel functions, have enrichment for NF-Y binding (pValue < 0.05, Table 1). For example, 19 of the 35 genes (54%) in the circadian rhythm module (#24) have NF-Y protein-bound NF-Y motif sites in their promoters, representing a 1.9 fold enrichment (pValue = 0.001) compared to the genome-wide average level. However, for modules #48, #62, #68, and #85, we observed under-representation for NF-YA/B binding in the ENCODE ChIP-Seq data (Table 1). Interestingly, module #48 functions in lipid metabolism specifically in adipocytes, while module #62 and #68 participate in developmental pattern regulation. Therefore, we hypothesize that the reason for low coverage in ENCODE ChIP-Seq data might be that the genes’ promoters in these modules are regulated by NF-Y TF in a cell type-specific manner. Consistent with this, the genes in these three modules are expressed at very low level in the three cell lines used in the ENCODE ChIP-Seq experiment (Supplementary Dataset 4).

Additionally, NF-Y TFs’ regulation on selected modules’ gene expression was also confirmed using published microarray data^25, 26^. Fleming *et al*. have conducted expression microarray analysis on HeLa S3 cell lines after depleting NF-YA gene’ expression using small hairpin RNA^25^. Based on their data, NF-YA’s depletion resulted in down-regulation of the cell cycle module (#1) and up-regulation of the nucleosome organization module (#10) and DNA damage response module (#42) (Supplementary Fig. S1). Benatti *et al*. also measured the transcriptomes of NF-YA depleted epithelial HCT116 cells, within which the modules involved in cholesterol biosynthesis (#7), pyruvate metabolism (#11), fatty acid oxidation (#49), and vesicle trafficking (#18) were repressed (Supplementary Fig. S1). As to our knowledge, NF-Y’s regulation on nucleosome organization (#10) and vesicle trafficking (#18) have not been reported before. It should be noted that there are three genes encoding NF-Y TFs in human, namely NF-YA, NF-YB, NF-YC, and knocking down just NF-YA might not affect all the modules regulated by the NF-Y motif described here.

### Gene expression modules regulated by the Sp1 motif

Sp1 is a ubiquitously expressed zinc finger TF that binds to the GC-rich Sp1 motif^29, 30^ (JASPAR motif ID: MA0079.3) and regulates diverse cellular processes such as cell differentiation and growth, apoptosis, immune response, DNA damage response, and chromatin remodeling. Polymorphisms in Sp1 binding motif sites are risk factors of many diseases such as osteoporosis, heart disease, type 2 diabetes, Alzheimer’s disease, and tumors^31–35^. The Sp1 binding sites have been mapped for human chromosome 21 and 22 using ChIP-Chip^36^. Additionally, the ENCODE project has mapped whole genome Sp1 binding sites in four human cell lines using ChIP-Seq. Interestingly, our network motif-based findings described below identified many novel Sp1-motif regulated genes that were not captured by the ChIP-Chip or the ENCODE ChIP-Seq experiments.

Among the genes used in our network analysis, 10,459 genes’ promoters contain the Sp1 motif. Our analysis identified 8,048 of them as potential Sp1 motif target genes. Among these target genes, 8,037 were identified by the motif position bias method (Z >= 4), 703 by the motif enrichment method (pValue <= 1E-4), and 694 were identified by both methods. The promoter sequences of all genes used in the analysis were then randomized and subjected to the same analysis. In each permutation run, on average only 3 genes were identified as Sp1 motif targets by our analysis. Thus the FDR for Sp1 motif analysis is 0.04% (3/8048).

**Figure 3:**
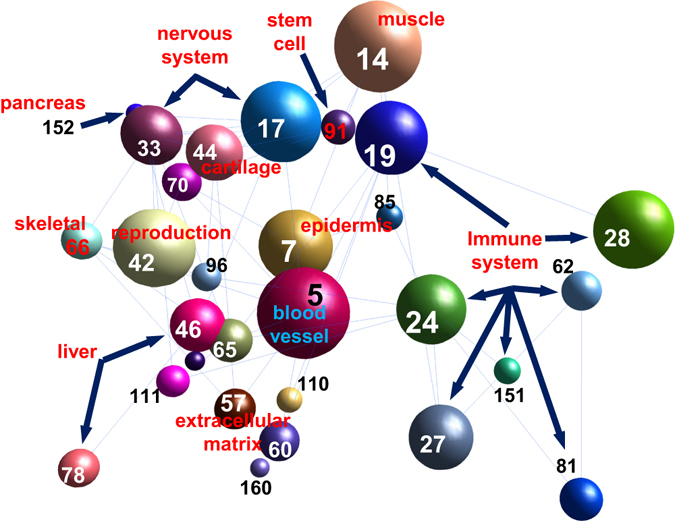
Co-expression modules regulated by the Sp1 motif. A sub-network for development and immune response modules regulated by the Sp1 motif is shown. The size of a sphere is proportional to the number of genes within the module. The number shown within the module represents module # shown in Table 2.

A sub-network extracted for the 8,048 Sp1 motif target genes contained 410 modules (Supplementary Dataset 5). Within these modules, the Sp1 motif shows position bias towards TSS in the genes’ promoters (Fig. 2d). 60 of these modules have significantly enriched GO terms (pValue < 5E-7) and can be divided into three categories: house-keeping or generic cellular function related modules, developmental related or tissue specific modules, and immunity related modules (Table 2). A sub-network for the immunity and development related modules is shown in Fig. 3. Consistent with previous reports on Sp1 motif functions, the immunity modules include platelet activation (module #19, 28), TNF-α signaling (#24), osteoclast differentiation (#27), interferon α/β signaling (#62), antigen processing and presentation via MHC I (#81), and chemokine-mediated signaling (#160) (Table 2)^37–41^

**Table 2:** Sp1 motif-regulated modules.

Module No.	Catergory	Tissue	# of genes in the Module	GO or Pathway Enrichment for the genes within the module			Binding of Sp1 protein to the gene promoters within the module according to ENCODE data			
				GO	GO/Pathway Enrichment pValue	Selected genes in the module with the enriched GO term	Genes with SP1 bound SP1 motif in promoter	Binding Enrichment Fold change	Binding Enrichmentp Value	Binding Depletion pValue
5	dev	blood vessel	81	angiogenesis	9.91E-15	NOS3/KDR/FLT1	9	0.27		1.49E-09
7	dev	skin	73	skin development	9.33E-12	TGM1/KLK7/KLK5	8	0.26		8.15E-09
14	dev	muscle	69	muscle system process	2.26E-36	NOS1/TNNI3/RYR1	14	0.49		1.42E-04
17	dev	nervous	61	synaptic transmission	5.19E-14	SLC6A3/TH/GRIN1	11	0.43		7.38E-05
33	dev	nervous	47	nervous system development	4.33E-17	KCNQ2/DCC/DLL1	7	0.36		7.50E-05
42	dev	reproduction	40	spermatogenesis	2.11E-10	DDX4/DDX25/SPO11	10	0.60		2.16E-02
44	dev	cartilage	39	connective tissue development	3.59E-10	COL2A1/COL11A2/COL10A1	9	0.55		1.21E-02
46	dev	liver	38	plasma lipoprotein particle assembly	1.38E-12	APOA1/APOB/APOA4	14	0.88		3.34E-01
57	dev	extracellular matrix	33	extracellular matrix organization	5.86E-14	MMP2/COL1A1/TNC	10	0.73		1.25E-01
60	dev	extracellular matrix	32	extracellular matrix organization	1.52E-09	PDGFRA/COL1A2/DCN	7	0.53		1.57E-02
65	dev		31	tissue morphogenesis	4.02E-09	HGF/IGFBP5/SFRP1	7	0.54		2.15E-02
66	dev	skeletal	31	anterior/posterior pattern specification	6.63E-16	HOXA10/HOXA1/EN1	3	0.23		1.04E-04
70	dev	nervous	29	glial cell differentiation	4.67E-09	S100B/ERBB3/RELN	1	0.08		3.47E-06
78	dev	liver	28	very-low-density lipoprotein particle clearance	4.40E-08	APOE/APOC3/APOC1	12	1.03	5.21E-01	
85	dev	skin	25	skin development	2.98E-10	SFN/ITGA3/KRT5	6	0.58		5.29E-02
91	dev	stem cell	24	stem cell maintenance	4.05E-07	NANOG/NODAL/LIN28A	10	1.00	5.77E-01	
96	dev		24	regionalization	1.66E-10	FOXC1/SIX1/TBX3	4	0.40		8.79E-03
110	dev		22	hemopoiesis	4.16E-07	SYK/HHEX/GATA2	8	0.87		3.92E-01
111	dev		22	pattern specification process	1.38E-10	PAX6/PBX1/NR2F2	5	0.55		5.30E-02
152	dev	pancreate	16	pancreatic A cell differentiation	8.89E-12	NEUROD1/INSM1/NKX2-2	1	0.15		2.23E-03
212	dev	thyroid	12	cellular modified amino acid metabolic process	7.22E-08	DUOX2/AHCY/DUOX1	2	0.40		6.71E-02
217	dev	nervous	12	negative regulation of glial cell differentiation	1.24E-09	FGFR3/ID2/ID4	4	0.80		3.92E-01
255	dev	reproduction	10	cilium assembly	2.02E-09	KIF3A/IFT88/FAM161A	6	1.44	1.95E-01	
272	dev	kidney	9	metanephros morphogenesis	2.47E-07	SMO/LGR4/FRAS1	3	0.80		4.41E-01
2	house		134	cell cycle	2.85E-69	BRCA1/BRCA2/BIRC5	94	1.68	1.85E-11	
4	house		100	cellular macromolecule metabolic process	3.44E-07	APC/ADD1/CSNK2A1	57	1.37	1.35E-03	
6	house		79	RNA metabolic process	5.65E-10	SP1/MTDH/PDPK1	47	1.43	1.01E-03	
9	house		72	pyruvate metabolic process	1.38E-12	ENO2/ENO1/DDIT4	33	1.10	2.72E-01	
15	house		66	regulation of macromolecule metabolic process	3.18E-13	MYC/JUN/EGR1	34	1.24	6.70E-02	
16	house		65	response to endoplasmic reticulum stress	3.73E-20	HSPA5/HSP90B1/PDIA3	51	1.88	1.43E-09	4.41E-01
23	house		56	protein folding	1.30E-22	HSP90AA1/HSPD1/HSPA8	36	1.54	5.14E-04	
26	house		55	translational elongation	2.74E-38	RPS19/RPL11/RPL5	41	1.79	7.19E-07	
31	house		48	RNA processing	2.44E-10	EIF4E/SRSF1/HNRNPD	32	1.60	4.00E-04	
32	house		47	lipid biosynthetic process	4.96E-26	SREBF1/FASN/PNPLA3	40	2.04	9.29E-10	
34	house		47	mRNA metabolic process	6.22E-09	PRMT5/PSMA6/RPS17	28	1.43	9.87E-03	
36	house		45	cellular response to DNA damage stimulus	4.84E-14	CDKN1A/MDM2/PCNA	28	1.49	4.24E-03	
37	house		44	histone modification	1.79E-13	MLL/EP300/CREBBP	27	1.47	6.48E-03	
38	house		42	cellular amino acid metabolic process	1.64E-17	ATF4/SLC7A5/ASS1	26	1.49	6.37E-03	
43	house		39	organic acid catabolic process	1.32E-12	MUT/HMGCL/BCKDHB	29	1.79	3.42E-05	
45	house		39	regulation of circadian rhythm	1.58E-08	PER2/PER3/PER1	29	1.79	3.42E-05	
48	house		37	ER to Golgi vesicle-mediated transport	1.69E-07	CREB3L2/USO1/SEC31A	23	1.49	9.39E-03	
67	house		31	fatty acid oxidation	5.18E-17	ACADM/HSD17B4/HADHA	17	1.32	9.61E-02	
79	house		28	pentose biosynthetic process	4.50E-12	G6PD/TALDO1/TKT	18	1.54	1.31E-02	
92	house		24	synapsis	6.30E-10	DMC1/STAG3/RNF212	9	0.90		4.23E-01
97	house		24	'de novo’ posttranslational protein folding	5.58E-11	TUBB3/TUBA1A/TUBB2B	10	1.00	5.77E-01	
105	house		22	ribonucleoprotein complex biogenesis	3.16E-10	NOLC1/TFB2M/WDR12	10	1.09	4.38E-01	
213	house		12	anaphase-promoting complex-dependent proteasomal ubiquitin-dependent protein catabolic process	3.13E-07	MAD2L2/PSMC3/UBE2S	8	1.60	7.24E-02	
19	immune		58	platelet activation	9.22E-11	ITGB3/CLU/F2R	15	0.62		9.05E-03
24	immune		55	immune response (TNFalpha)*	1.85E-17	TNF/IL1B/NFKB1	29	1.27	6.35E-02	
27	immune		54	immune system process (Osteoclast differentiation)*	2.36E-14	ITGB2/CD4/FCGR3B	17	0.76		8.20E-02
28	immune		54	platelet activation	3.46E-09	ILK/RAP1A/CFL1	37	1.64	5.83E-05	
62	immune		32	defense response to virus (Interferon alpha/beta signaling)*	5.24E-16	PML/EIF2AK2/BST2	10	0.75		1.55E-01
81	immune		27	antigen processing and presentation of peptide antigen via MHC class I	1.95E-14	HLA-B/TAP1/PSMB9	9	0.80		2.50E-01
151	immune		16	positive regulation of alpha-beta T cell proliferation	2.95E-07	IL12B/IL23A/EBI3	10	1.50	7.61E-02	
160	immune		16	chemokine-mediated signaling pathway	7.80E-08	CCR5/CCR6/CXCR6	2	0.30		1.32E-02
30	other		49	regulation of transcription, DNA-templated	9.71E-30	ZNF267/ZNF420/ZNF92	35	1.71	2.29E-05	
73	other		29	cellular lipid metabolic process	3.70E-09	ALOX15B/FA2H/CRAT	7	0.58		3.91E-02
269	other		10	negative regulation of MAP kinase activity	1.22E-12	SPRY2/DUSP6/SPRED1	2	0.48		1.42E-01
287	other		9	JAK-STAT cascade involved in growth hormone signaling pathway	2.12E-07	STAT3/STAT5A/STAT5B	4	1.07	5.59E-01	
349	other		6	regulation of transposition	5.01E-10	APOBEC3G/APOBEC3C/APOBEC3B	3	1.20	4.90E-01	

Note: * denotes enriched pathways.

.

The house-keeping or generic cellular function category contains modules with known functions of Sp1 such as cell cycle regulation (module #2), DNA damage response and DNA repair (#36), response to stimulus (#15), chromatin modification (#37), and lipid biosynthesis (#32) (Table 2)^30, 42^. It also includes novel functional modules regulated by Sp1: RNA processing (#6, 31, 34), protein folding (#23), vesicle trafficking (#48), and regulation of circadian rhythm (#45) (Table 2).

**Figure 4:**
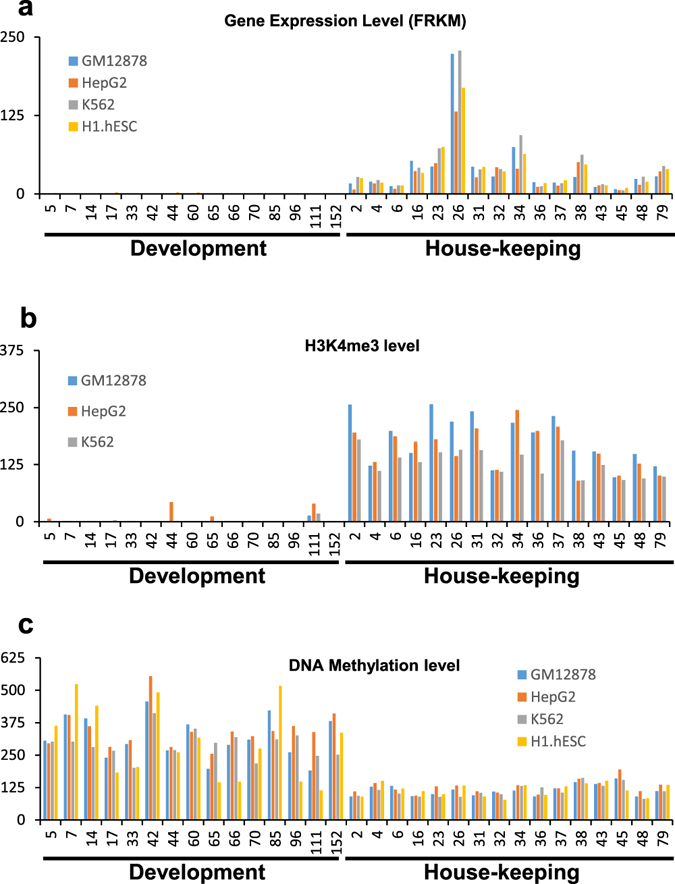
Gene expression and epigenetic regulation of Sp1 motif regulated modules. (**a**) The median gene expression level for genes within the Sp1-regulated modules in four human cell lines. The module numbers shown in X-axis are related to development and house-keeping categories described in Table 2. FPKM values from RNA-Seq experiments conducted by the ENCODE project is used as Y-axis. (**b**) The median H3K4me3 level in the promoters for genes within the Sp1-regulated modules in three human cell lines used in the ENCODE project. (**c**) The average promoter DNA methylation level for the genes within the Sp1-regulated modules in four human cell lines used in the ENCODE project.

The ENCODE ChIP-Seq data of four human cell lines (K562, GM12878, H1-hESC, and HepG2) identified 4,361 gene promoters with Sp1 motif bound by Sp1 TF among all 10,459 Sp1 motif-containing genes used in our network. Out of the 23 house-keeping modules identified in our analyses, 16 show enrichment for Sp1 TF binding in the ENCODE ChIP-Seq data (pValue < 0.05, Table 2). These results provide validation of our network findings. Genes within these 16 modules are expressed well in the four cell lines used in the ENCODE project (Fig. 4a) and in diverse human primary cell lines (Supplementary Fig. S2).

The development-related or tissue specificity-related functions category regulated by Sp1 includes various modules functioning in development of the nervous system (#17, 33, 70, 217), skeletal system (#66), muscle (#14), skin (#7, 85), hepatocyte (#46), blood vessel (#5), pancreas (#152), thyroid (#212), kidney (#272), cartilage (#44), reproduction systems (#42, 255), and stem cell (#91) (Fig. 3 and Table 2). Previous studies have shown Sp1’s involvement in the development of these tissues individually. For example, Sp1 is important for nervous system development^43^. Huntington’s disease, a neurodegenerative disease, is caused by mutated Huntington protein that interacts with Sp1 and thus fails to bind to DNA^43^. Sp1 also regulates the expression of *NOS3* gene in the module #5 that encodes the endothelial nitric oxide synthase critical for blood vessel and embryonic heart development^44^. In the skin development module (#7), Sp1 functions as a repressor to down-regulate *KLK5* and *KLK7* expression^45^. Additional examples of Sp1 motif regulating developmental genes are shown in Supplementary Dataset 6, which together verify our novel network findings.

Interestingly, when we looked into the ENCODE ChIP-Seq data, the Sp1 motif sites within the genes of above-described developmental modules are under-represented or even depleted of Sp1 TF binding in the four human cell lines used for ChIP-Seq analyses (Table 2). Compared to Sp1 regulated house-keeping modules, the developmental modules have little or no expression in the four cell lines used in the ENCODE project (Fig. 4a). Instead, they display specific expression in other primary cell lines (Supplementary Fig. S2), indicating cell/tissue specific expression of genes regulated by the Sp1 motif.

### Epigenetic regulation of Sp1-regulated house-keeping and the developmental modules

We reasoned that the expression difference between the Sp1-regulated house-keeping modules and the developmental modules could be due to epigenetic regulation. Therefore, we analyzed histone H3 lysine 4 tri-methylation (H3K4me3) and DNA methylation patterns for the promoters of target genes identified in our analyses using data from ENCODE. Apart from TF binding data, the ENCODE project has also measured these two epigenetic marks over ~60 different cell lines that includes the ones used for the Sp1 ChIP-Seq experiments (http://genome.ucsc.edu/cgi-bin/hgTrackUi?db=hg19&g=wgEncodeUwHistone; http://genome.ucsc.edu/cgi-bin/hgTrackUi?db=hg19&g=wgEncodeHaibMethyl450).

H3K4me3 is normally associated with active or poised promoters^46^. All house-keeping modules identified in our analyses show high levels of H3K4me3 (Fig. 4b) in K562, GM12878 and HepG2 human cell lines (data for H1-hESC are not available), consistent with their high expression levels (Fig. 4a). Interestingly, in agreement with their low gene expression levels, most of the developmental modules (Fig. 4a) have low H3K4me3 levels in these three cell lines (Fig. 4b) as well as in 56 other cell lines (Supplementary Fig. S3) used by the ENCODE project. However, there are cell-line specific H3K4me3 level hikes for many of the Sp1-regulated developmental modules that match their functions. For example, module #85 is enriched with epithelium development related genes and their H3K4me3 levels are relatively higher in esophageal epithelial cells (HEEpiC), mammary epithelial cells (HMEC), and small airway epithelial cells (SAEC) (Supplementary Fig. S3). Module #5, enriched with blood vessels genes, has highest H3K4me3 levels in umbilical vein endothelial cells (HUVEC) (Supplementary Fig. S3).

DNA methylation in gene promoters normally represses their transcription. Consistent with this, we found higher methylation levels in developmental gene modules than house-keeping modules regulated by Sp1 in the four-cell lines used in the ENCODE ChIP-Seq (Fig. 4c), as well as in 59 other cell lines used in ENCODE (Supplementary Fig. S3). Cell-line specific reduction in methylation level is also observed in the developmental modules. For example, the lowest DNA methylation level for module #85 is in three epithelium-related cell lines NHBE (bronchial epithelial cells), HEEpiC, and SAEC, and for module #5 in the HUVEC cells (Supplementary Fig. S3). Therefore, we hypothesize that Sp1 TF only binds to the gene promoters of the developmental modules after they are activated in specific cells/tissues through epigenetic modifications, and thus Sp1 regulates genes in a cell/tissue-specific manner. Consistent with this, Sp1 binds to the promoter of *NOS3* and *ACVRL1* of module #5 specifically in the endothelial HUVEC cell line, and in other cell lines after treatment with DNA methylation inhibitors^44, 47^. DNA methylation also regulates the expression of *LDHC* of module #28^48^.

### Gene expression modules regulated by other motifs

We also analyzed other human TF motifs catalogued in the JASPAR database or derived from the ENCODE project^1, 49^. Gene targets for 37 motifs with FDR range from 0.1% to 4.2% were identified (Supplementary Dataset 7). Discussed below are three specific examples of gene modules regulated by Yin Yang (YY1), Regulatory Factor X 2 (RFX2), and Interferon Regulatory Factor 1 (IRF1) motifs (Table 3).

**Table 3:** Gene modules regulated by YY1, RFX2, and IRF1 motifs.

Motif	Module No.	# of genes in the Module	Enriched GO/Pathway	GO/Pathway Enrichment pValue	Selected Genes with the enriched GO term
YY1	1	47	cellular respiration	1.97E-28	SDHB/NDUFS4/NDUFS3
YY1	4	31	mitotic cell cycle	5.62E-14	RRM1/NDC80/RACGAP1
YY1	7	27	RNA processing	2.41E-10	RBM8A/RBM4/CNOT3
YY1	10	25	translational elongation	3.34E-27	RPSA/RPS3/RPL13A
YY1	12	22	fatty acid beta-oxidation	1.13E-06	HADHA/ACADVL/HADHB
YY1	17	17	ribosome biogenesis	3.81E-06	NOLC1/NIP7/CIRH1A
YY1	18	17	histone modification	2.60E-11	EP300/CREBBP/CTCF
YY1	37	11	RNA metabolic process	5.15E-06	PER1/POLR2A/PHF8
YY1	42	10	RNA splicing	6.86E-11	TARDBP/SRSF1/DDX39
YY1	43	10	type I interferon signaling pathway	8.29E-06	IRF1/ADAR/XAF1
YY1	46	9	regulation of mRNA processing	4.14E-06	CWC22/IWS1/CCDC55
RFX2	1	73	cilium morphogenesis	9.10E-10	ZMYND10/FOXJ1/AK7
RFX2	2	48	cilium assembly	4.00E-19	AHI1/CBY1/BBS1
RFX2	4	20	protein folding	1.03E-11	HSP90AA1/HSP90B1/HSPA1L
RFX2	5	14	microtubule-based movement	1.32E-06	KIF14/KIF23/KIF18A
RFX2	10	12	respiratory electron transport chain	4.77E-09	NDUFS3/CYC1/NDUFA5
RFX2	21	8	synaptic transmission	5.03E-08	CAMK2A/KCNIP2/SLC17A7
RFX2	33	6	regulation of striated muscle cell differentiation	1.13E-06	AKAP6/SMYD1/KBTBD10
RFX2	42	5	mitotic cell cycle	5.34E-07	PRKDC/MCM7/CENPH
IRF1	1	79	Interferon alpha/beta signaling	2.92E-30	STAT1/MX1/ADAR
IRF1	2	60	antigen processing and presentation of peptide antigen via MHC class I	3.98E-23	HLA-B/HLA-A/HLA-C
IRF1	3	35	Interferon gamma signaling	9.26E-07	JAK2/CXCL10/IL12RB1
IRF1	4	34	response to virus	3.44E-09	CCL5/IFNB1/IL12A
IRF1	5	33	synaptic transmission	1.00E-06	HTR2C/SNAP25/GABRG2
IRF1	6	33	hair follicle development	8.11E-06	NGFR/TFAP2C/SOSTDC1
IRF1	7	31	leukocyte activation	3.80E-10	TLR1/ICOS/CD247
IRF1	12	12	IFN alpha signaling	3.12E-27	IFNA17/IFNA5/IFNW1
IRF1	13	10	NOD-like receptor signaling pathway	6.88E-06	TLR2/NOD2/CASP1
IRF1	18	7	regulation of transposition	4.35E-13	APOBEC3G/APOBEC3F/APOBEC3C
IRF1	20	6	neuronal action potential	3.00E-06	PLP1/ASPA/SCN7A

YY1 is a zinc finger TF with both activation and repression functions^50^. The modules identified for YY1 motif (MA0095.2) regulate two mitochondria pathways (module #1 for cellular respiration, and #12 for fatty acid beta-oxidation), cell cycle (#4), histone modification (#18), RNA processing, splicing and metabolism (#7, 37, 42, 46), ribosome biogenesis (#17), translational elongation (#10), and type I interferon signaling pathway (#43). Previous reports identified YY1 binding targets enriched with mitochondria, ribosomal, and RNA metabolism genes^51–53^, which confirm our network findings.

The RFX TFs belong to a winged-helix DNA-binding domain-containing TF family conserved in yeast, flies, and vertebrates^54^. They play important roles in transcriptional regulation of ciliogenesis^55^. We identified gene co-expression modules regulated by the human RFX2 promoter motif (MA0600.1). Among them are three modules involved in cilium morphogenesis, cilium assembly, and microtubule-based movement (module #1, 2, 14). Other modules indicate novel processes regulated by RFX2 or RFX TFs, such as synaptic transmission (#21), striated muscle cell differentiation (#33), mitotic cell cycle (#42), cellular respiration (#10), and protein folding (#4). Supporting our findings, RFX2 is involved in nerve cell response to paclitaxel in rats, while the RFX TF gene *sak* + regulates mitotic cell cycle in fission yeast^56, 57^.

The IRF family TFs are important regulators of immunity^58^. The gene co-expression modules regulated by the human IRF1 promoter motif (MA0050.2) include those for Interferon α/β signaling (module #1, #12), interferon γ signaling (#3), antigen processing and presentation via MHC I (#2), NOD-like receptor signaling (#13), response to virus (#4), leukocyte activation (#7), and regulation of transposition (#18). Interestingly, also included are three developmental related modules, for synaptic transmission (#5), neuronal action potential (#20), and hair follicle development (#6), indicating that IRF1 or related IRF TFs might regulate these processes.

The motif target lists of NF-Y, Sp1, YY1, RFX2, and IRF1 from our analyses described here together with the target list of other 34 motifs listed in Supplementary Dataset 7, provide the basis for deciphering the human gene expression regulatory mechanisms that shape the expression landscape as captured by our gene co-expression network.

## Discussion

We describe here a human gene co-expression network that we used to identify gene co-expression modules regulated by various *cis*-regulatory motifs. Compared to other co-expression network studies, a distinctive advantage of our approach is that it provides in-depth characterizations of the TF motifs that regulate gene expression within the network. Using motif enrichment and, more importantly, motif position bias methods, many target genes were identified with high confidence for well-studied NF-Y, Sp1, and other TF motifs. Interestingly, ~90% of the Sp1 motif targets were only identified by the position bias method but not by the typically used motif enrichment method. Additionally, rather than merely producing a list of target genes for selected motifs, our analysis also organized and placed them into diverse gene co-expression modules, providing a clear and streamlined overview of the gene expression landscapes regulated by specific motifs.

The gene network and gene co-expression modules also enable easy integration of various types of omics-based data into a coherent regulatory system. While the regulatory modules were identified based on gene expression and promoter sequence analysis, we used independent TF-promoter interaction data from the ENCODE ChIP-Seq experiments to verify our prediction. The modules from our analyses can be interrogated with gene expression and various types of epigenomic data. For example, the Sp1 motif target genes’ profiles on H3K4me3 and DNA methylation show perfect correlation between the Sp1 binding profile and gene expression profile in different cell types. Furthermore, we hypothesize that the dynamic change in these two epigenetic marks will be accompanied by dynamic change in Sp1 binding and its target genes’ expression, which provides a possible mechanism on how Sp1 differentially regulates house-keeping and tissue specific gene co-expression modules. Cell lines-specific ChIP-Seq or ChIP-qPCR measuring the SP1 binding sites will be helpful to validate such a hypothesis. The modules could also be used to dissect the function of other epigenetic marks on gene expression regulation, including the genome-wide data for more than 20 histone marks deposited by the NIH Roadmap Epigenome project^59^.

The robustness and novelty of our approach is demonstrated by the gene co-expression modules identified in our analyses. For example, our analyses captured known and novel modules for the two well-studied motifs NF-Y and Sp1. Since these two motifs are widely distributed in the genome, it makes it hard to identify their targets *via* typically used motif enrichment method. Therefore, our motif position bias method towards TSS made it possible to identify targets of NF-Y, Sp1 and other TFs. While most of the NF-Y targets are related to house-keeping functions, the Sp1 targets do include immunity and tissue development processes. The large number of target modules regulated through Sp1 motif is also reflected by the recognition that Sp1 target gene expression deregulation is associated with many disease risk factors. These deregulations usually involve polymorphism within Sp1 motif sites of target gene promoters. We expect that our network described here with its expression module based approach will allow for and promote the identification of additional disease-associated deregulation incidents.

**Figure 5:**
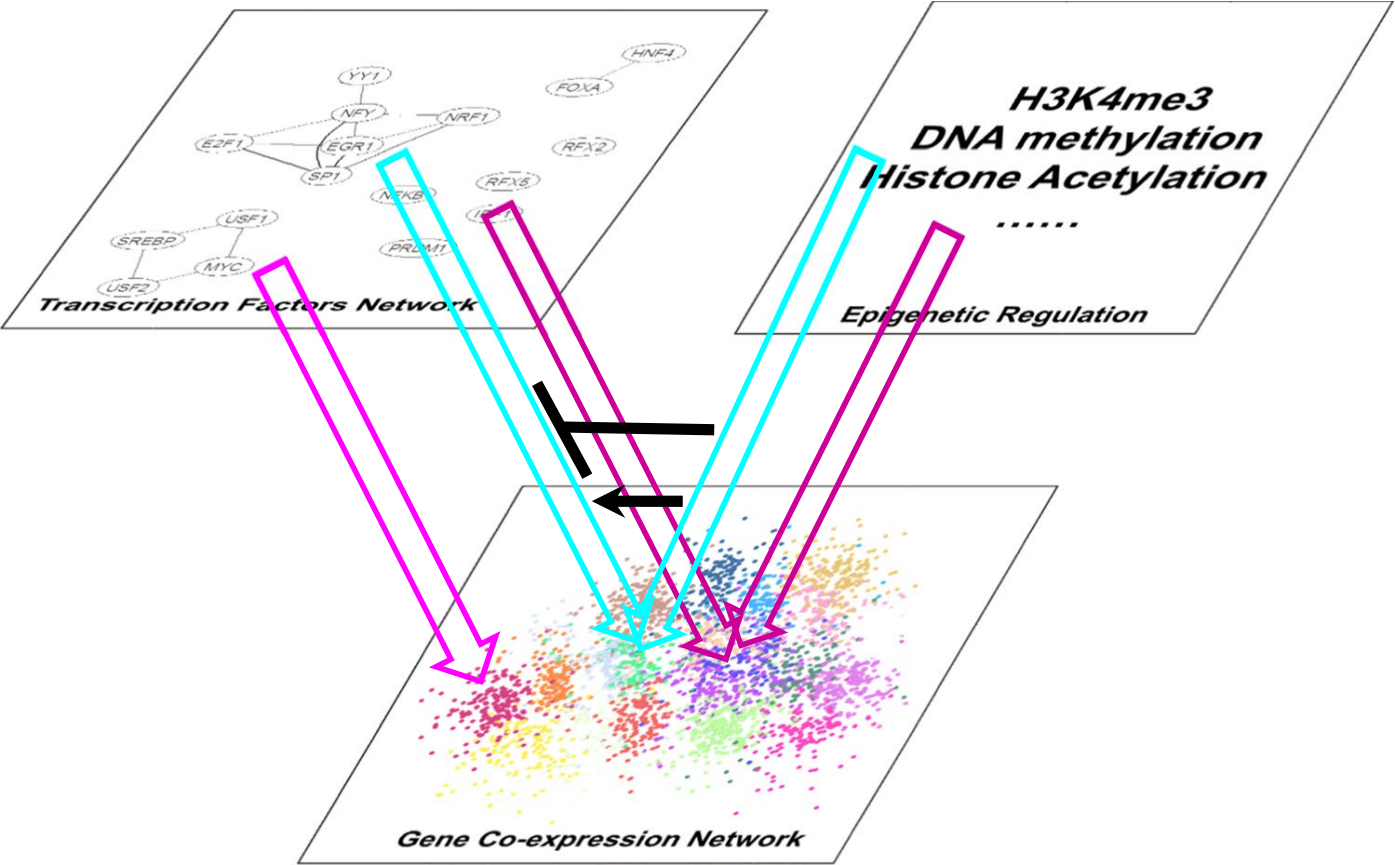
A gene regulatory system with different layers of regulations. The gene regulatory system consists of three layers: gene co-expression network, transcription factors network, and epigenetic regulation. Gene co-expression network capture the output of the regulatory system, i.e. the expression patterns, which are regulated by both transcription factors network and epigenetic regulation.

Our results also show that integrating gene co-expression network with different types of omics data allows construction of integrated gene expression systems with multiple layers of regulation (Fig. 5). The bottom layer of such an integrated approach will conceivably be the gene co-expression network, where co-expression modules can be identified. These co-expression modules perform specific functions in metabolism or signaling pathways. Superimposed exists a layer of epigenetic regulation affecting promoter activation, through H3K4me3, DNA methylation, histone acetylation, and other processes. It should be noted that the current study was mainly focused on promoter motifs proximal to the TSS, but there are also other *cis*-regulatory motifs located at enhancers more than 2.5 kb upstream or 0.2 kb downstream of the TSS. Datasets generated by the chromosome conformation capture techniques would be helpful to incorporate such distal regulation into our current model. These regulations can determine the activated or deactivated status of gene promoters. Another parallel layer is represented by different TFs, which will bind to *cis*-regulatory motifs within the activated gene promoters to regulate their expression. The TFs themselves can interact with each other to co-regulate their downstream target genes. Therefore, the results described here provide a snapshot at the identification of gene co-expression modules (expression layer), motif-promoter interactions, and epigenetic regulatory effects. However, it must be noted that multiple TFs, of the same or different gene families, may bind to same motif sites within a gene’s promoter. Therefore, in the future identification of corresponding TF that drive the expression of the individual modules will be an important task.

## Methods

### Gene co-expression network

Publicly available microarray datasets generated with Affymetrix U133 plus 2.0 arrays and deposited in the ArrayExpress database^13^ were used to construct the human gene co-expression network based on GGM as described previously^14^. See the supplementary methods for more details.

### Promoter motif analysis and target gene identification

Promoter sequences for the 19,718 analyzed genes were extracted as 2500 bp upstream of TSS and 200 bp downstream of TSS, except for NF-Y motif analysis. For NF-Y motif, due to its high prevalence, promoter sequences are defined as 1000 bp upstream of TSS. After excluding the repeat sequences and transposon sequences (by replacing all nucleotides within these sequences with the letter code “N”), the promoter sequences were scanned for presences of selected motif sites with motif position weight matrix (PWM) obtained from JASPAR, or from those motifs derived from the ENCODE project^1, 49^, using POSSUM with a pValue cut off at 4^−8^ ^60, 61^. Motif enrichment and motif position bias analysis were then carried out for genes in the network to identify target genes regulated by the motif. Permutation analysis on randomized promoters was conducted to assess the FDR. See supplementary experimental procedure for more details.

Briefly, the motif position bias analysis calculates the extent a motif’s distribution deviating from random uniform distribution towards TSS within a group of promoters. A background model for a uniformly distributed motif is first established to calculate the motif’s expected distance from TSS and its variance. Suppose for all promoters in the group, defined as *M* bps upstream and *N* bps downstream of TSS, there are *K* free bps in total that are not occupied by repeat or transposon sequences. The motif has equal chance to appear in any of these *K* bp. Suppose in position *i* (−*M* <= *i* <= *N*) along the promoters, which is *i* bp relative to TSS, there are *k*_*i*_ free bps, i.e. there are *k*_*i*_ promoters are not occupied by repeat or transposon sequences in that position. Then, the motif’s expected mean distance from TSS is given by:

2 $$E(d)=\,\sum _{i\,=-M}^{N}\frac{{k}_{i}}{K}\times |i|$$

And its variance is given by:

3 $$V(d)=\,\sum _{i\,=-M}^{N}\frac{{k}_{i}}{K}\times {i}^{2}-{(E(d))}^{2}$$

For an actual motif appears *n* times in that group of promoters, with mean distance $\overline{|x|}$ from the TSS, a Z score is calculated as:

4 $$Z=\,\frac{E(d)-\overline{|x|}\,}{\sqrt{\frac{V(d)}{n}}}$$

A Z score larger or equal to selected cutoff value is considered to have significant position bias towards TSS.

### Co-expression modules GO, TF binding, H3K4me3, methylation, RNA-Seq data, and NF-Y related microarray data analyses

Target genes for a specific motif were used to extract a sub-network from the whole human gene co-expression network. The sub-network was clustered using the MCL clustering algorithm and visualized using BioLayout Express 3D^62, 63^. GO analysis was carried out using the GOStats package in Bioconductor^64^.

To evaluate the enrichment of corresponding TF’s binding to its motifs within the promoters of co-expression modules, the ENCODE TF binding data were used^3^. RNA-Seq, H3K4me3, and DNA methylation (methyl 450 K beads) from the ENCODE project were used to evaluate expression and epigenetic modification level associated with the genes in the modules. See supplementary experimental procedure for more details.

Two published microarray datasets^25, 26^ (GSE40215 and GSE70543) were also used to assess if NF-Y motif modules were regulated by the NF-Y TFs. Within these two dataset, the NF-YA gene was knocked down in using small hairpin RNA in two human cell lines, HeLa S3 and HCT116^25, 26^. The list of gene regulation values from each dataset was analyzed with the Gene Set Enrichment Analysis (GSEA v2.2.0)^65^ to see if any of the NF-Y motif modules were up- or down-regulated within the sample. A module was called up-regulated if its NES value was >0 and FDR value <= 0.05, or down-regulated if its NES value was <0 and FDR value <= 0.05.

## Electronic supplementary material

Supplementary methods and figures and datasets

Supplementary Dataset 1

Supplementary Dataset 2

Supplementary Dataset 3

Supplementary Dataset 4

Supplementary Dataset 5

Supplementary Dataset 6

Supplementary Dataset 7

Supplementary Dataset 8

Supplementary Dataset 9

Supplementary Dataset 10

## References

[1] Mathelier A (2014). JASPAR 2014: an extensively expanded and updated open-access database of transcription factor binding profiles. Nucleic acids research.

[2] Wingender E, Dietze P, Karas H, Knuppel R (1996). TRANSFAC: a database on transcription factors and their DNA binding sites. Nucleic acids research.

[3] Encode PC (2012). An integrated encyclopedia of DNA elements in the human genome. Nature.

[4] Gerstein MB (2012). Architecture of the human regulatory network derived from ENCODE data. Nature.

[5] Neph S (2012). An expansive human regulatory lexicon encoded in transcription factor footprints. Nature.

[6] Zhang B, Horvath S (2005). A general framework for weighted gene co-expression network analysis. Stat Appl Genet Mol Biol.

[7] Fantom Consortium, the, R. P. & Clst. A promoter-level mammalian expression atlas. *Nature* **507**, 462–470, doi:10.1038/nature13182 (2014). 10.1038/nature13182 PMC4529748 24670764

[8] Mabbott NA, Baillie JK, Brown H, Freeman TC, Hume DA (2013). An expression atlas of human primary cells: inference of gene function from coexpression networks. BMC genomics.

[9] Oldham MC (2008). Functional organization of the transcriptome in human brain. Nature neuroscience.

[10] Parikshak NN (2013). Integrative functional genomic analyses implicate specific molecular pathways and circuits in autism. Cell.

[11] Saris, C. G. J. *et al*. Weighted gene co-expression network analysis of the peripheral blood from Amyotrophic Lateral Sclerosis patients. *BMC genomics* **10**, doi:10.1186/1471-2164-10-405 (2009). 10.1186/1471-2164-10-405 PMC2743717 19712483

[12] Ma S, Shah S, Bohnert HJ, Snyder M, Dinesh-Kumar SP (2013). Incorporating motif analysis into gene co-expression networks reveals novel modular expression pattern and new signaling pathways. PLoS Genet.

[13] Rustici G (2013). ArrayExpress update–trends in database growth and links to data analysis tools. Nucleic acids research.

[14] Ma S, Gong Q, Bohnert HJ (2007). An Arabidopsis gene network based on the graphical Gaussian model. Genome research.

[15] Schäfer, J. & Strimmer, K. A shrinkage approach to large-scale covariance matrix estimation and implications for functional genomics. *Stat Appl Genet Mol Biol* **4**, Article32, doi:10.2202/1544-6115.1175 [doi] (2005). 10.2202/1544-6115.1175 16646851

[16] de la Fuente A, Bing N, Hoeschele I, Mendes P (2004). Discovery of meaningful associations in genomic data using partial correlation coefficients. Bioinformatics.

[17] van Dongen, S. *Graph clustering by flow simulation*, University of Utrecht, (2000).

[18] Linhart C, Halperin Y, Shamir R (2008). Transcription factor and microRNA motif discovery: the Amadeus platform and a compendium of metazoan target sets. Genome research.

[19] Tompa M (2005). Assessing computational tools for the discovery of transcription factor binding sites. Nat Biotechnol.

[20] Sinha S, Tompa M (2002). Discovery of novel transcription factor binding sites by statistical overrepresentation. Nucleic acids research.

[21] Elemento O, Slonim N, Tavazoie S (2007). A universal framework for regulatory element discovery across all genomes and data types. Mol Cell.

[22] Yokoyama KD, Ohler U, Wray GA (2009). Measuring spatial preferences at fine-scale resolution identifies known and novel cis-regulatory element candidates and functional motif-pair relationships. Nucleic acids research.

[23] Mantovani R (1998). A survey of 178 NF-Y binding CCAAT boxes. Nucleic Acids Res.

[24] Dolfini D, Gatta R, Mantovani R (2012). NF-Y and the transcriptional activation of CCAAT promoters. Critical reviews in biochemistry and molecular biology.

[25] Fleming JD (2013). NF-Y coassociates with FOS at promoters, enhancers, repetitive elements, and inactive chromatin regions, and is stereo-positioned with growth-controlling transcription factors. Genome Res.

[26] Benatti P (2016). NF-Y activates genes of metabolic pathways altered in cancer cells. Oncotarget.

[27] Smith J, Mowla S, Prince S (2011). Basal transcription of the human TBX3 gene, a key developmental regulator which is overexpressed in several cancers, requires functional NF-Y and Sp1 sites. Gene.

[28] Wang Y, Stary JM, Wilhelm JE, Newmark PA (2010). A functional genomic screen in planarians identifies novel regulators of germ cell development. Genes Dev.

[29] Dynan WS, Tjian R (1983). The promoter-specific transcription factor Sp1 binds to upstream sequences in the SV40 early promoter. Cell.

[30] Wierstra I (2008). Sp1: emerging roles–beyond constitutive activation of TATA-less housekeeping genes. Biochemical and biophysical research communications.

[31] Bond GL (2004). A single nucleotide polymorphism in the MDM2 promoter attenuates the p53 tumor suppressor pathway and accelerates tumor formation in humans. Cell.

[32] Grant SF (1996). Reduced bone density and osteoporosis associated with a polymorphic Sp1 binding site in the collagen type I alpha 1 gene. Nature genetics.

[33] Maloney B (2010). Functional Characterization of Three Single-Nucleotide Polymorphisms Present in the Human APOE Promoter Sequence: Differential Effects in Neuronal Cells and on DNA-Protein Interactions. American Journal of Medical Genetics Part B-Neuropsychiatric Genetics.

[34] Osawa H (2004). The G/G genotype of a resistin single-nucleotide polymorphism at -420 increases type 2 diabetes mellitus susceptibility by inducing promoter activity through specific binding of Sp1/3. American journal of human genetics.

[35] Schou J (2012). Genetic Variation in ABCG1 and Risk of Myocardial Infarction and Ischemic Heart Disease. Arteriosclerosis Thrombosis and Vascular Biology.

[36] Cawley S (2004). Unbiased mapping of transcription factor binding sites along human chromosomes 21 and 22 points to widespread regulation of noncoding RNAs. Cell.

[37] Hauses M, Tonjes RR, Grez M (1998). The transcription factor Sp1 regulates the myeloid-specific expression of the human hematopoietic cell kinase (HCK) gene through binding to two adjacent GC boxes within the HCK promoter-proximal region. The Journal of biological chemistry.

[38] Falvo JV (2000). Stimulus-specific assembly of enhancer complexes on the tumor necrosis factor alpha gene promoter. Molecular and cellular biology.

[39] Mangan JK (2004). Mechanisms associated with IL-6-induced up-regulation of Jak3 and its role in monocytic differentiation. Blood.

[40] Tone M, Tone Y, Babik JM, Lin CY, Waldmann H (2002). The role of Sp1 and NF-kappa B in regulating CD40 gene expression. The Journal of biological chemistry.

[41] Santiago FS, Khachigian LM (2004). Ets-1 stimulates platelet-derived growth factor A-chain gene transcription and vascular smooth muscle cell growth via cooperative interactions with Sp1. Circulation research.

[42] Li L, Davie JR (2010). The role of Sp1 and Sp3 in normal and cancer cell biology. Annals of anatomy = Anatomischer Anzeiger: official organ of the Anatomische Gesellschaft.

[43] Dunah AW (2002). Sp1 and TAFII130 transcriptional activity disrupted in early Huntington’s disease. Science.

[44] Chan Y (2004). The cell-specific expression of endothelial nitric-oxide synthase: a role for DNA methylation. The Journal of biological chemistry.

[45] Bin L, Kim BE, Hall CF, Leach SM, Leung DY (2011). Inhibition of transcription factor specificity protein 1 alters the gene expression profile of keratinocytes leading to upregulation of kallikrein-related peptidases and thymic stromal lymphopoietin. The Journal of investigative dermatology.

[46] Vermeulen M, Timmers HT (2010). Grasping trimethylation of histone H3 at lysine 4. Epigenomics.

[47] Garrido-Martin EM (2010). Characterization of the human Activin-A receptor type II-like kinase 1 (ACVRL1) promoter and its regulation by Sp1. BMC molecular biology.

[48] Tang H, Goldberg E (2009). Homo sapiens lactate dehydrogenase c (Ldhc) gene expression in cancer cells is regulated by transcription factor Sp1, CREB, and CpG island methylation. Journal of andrology.

[49] Kheradpour P, Kellis M (2014). Systematic discovery and characterization of regulatory motifs in ENCODE TF binding experiments. Nucleic acids research.

[50] Shi Y, Seto E, Chang LS, Shenk T (1991). Transcriptional repression by YY1, a human GLI-Kruppel-related protein, and relief of repression by adenovirus E1A protein. Cell.

[51] Cunningham JT (2007). mTOR controls mitochondrial oxidative function through a YY1-PGC-1alpha transcriptional complex. Nature.

[52] Xi H (2007). Analysis of overrepresented motifs in human core promoters reveals dual regulatory roles of YY1. Genome research.

[53] Lu L (2013). Genome-wide survey by ChIP-seq reveals YY1 regulation of lincRNAs in skeletal myogenesis. The EMBO journal.

[54] Emery P, Durand B, Mach B, Reith W (1996). RFX proteins, a novel family of DNA binding proteins conserved in the eukaryotic kingdom. Nucleic acids research.

[55] Choksi SP, Lauter G, Swoboda P, Roy S (2014). Switching on cilia: transcriptional networks regulating ciliogenesis. Development.

[56] Wheeler HE (2013). Integration of cell line and clinical trial genome-wide analyses supports a polygenic architecture of Paclitaxel-induced sensory peripheral neuropathy. Clinical cancer research: an official journal of the American Association for Cancer Research.

[57] Wu SY, McLeod M (1995). The sak1+ gene of Schizosaccharomyces pombe encodes an RFX family DNA-binding protein that positively regulates cyclic AMP-dependent protein kinase-mediated exit from the mitotic cell cycle. Molecular and cellular biology.

[58] Taniguchi T, Ogasawara K, Takaoka A, Tanaka N (2001). IRF family of transcription factors as regulators of host defense. Annu. Rev. Immunol..

[59] Bernstein BE (2010). The NIH Roadmap Epigenomics Mapping Consortium. Nature biotechnology.

[60] Touzet H, Varre JS (2007). Efficient and accurate P-value computation for Position Weight Matrices. Algorithms for molecular biology: AMB.

[61] Fu Y, Frith MC, Haverty PM, Weng Z (2004). MotifViz: an analysis and visualization tool for motif discovery. Nucleic acids research.

[62] Enright AJ, Van Dongen S, Ouzounis CA (2002). An efficient algorithm for large-scale detection of protein families. Nucleic acids research.

[63] Theocharidis A, van Dongen S, Enright AJ, Freeman TC (2009). Network visualization and analysis of gene expression data using BioLayout Express(3D). Nat Protoc.

[64] Falcon S, Gentleman R (2007). Using GOstats to test gene lists for GO term association. Bioinformatics.

[65] Subramanian A (2005). Gene set enrichment analysis: a knowledge-based approach for interpreting genome-wide expression profiles. Proc Natl Acad Sci USA.

