# Supplementary material for: Discovery of Novel Human Gene Regulatory Modules from Gene Co-expression and Promoter Motif Analysis
Source: Sci Rep. 2017 Jul 17;7:5557. doi: 10.1038/s41598-017-05705-2 (PMC5514134; doi:10.1038/s41598-017-05705-2)
Supplement: Supplementary file 1 — Supplementary methods and figures and datasets [file 41598_2017_5705_MOESM1_ESM.pdf]

# Discovery of Novel Human Gene Regulatory Modules from Gene Co-expression and Promoter Motif Analysis

Shisong Ma[1,2*], Michael Snyder[3], and Savithramma P Dinesh-Kumar[2*]

[1]School of Life Sciences, University of Science and Technology of China, Hefei, Anhui, 230027, China

[2]Department of Plant Biology and the Genome Center, College of Biological Sciences, University of California, Davis, CA 95616.

[3]Department of Genetics, Stanford University, Stanford, CA 94305.

*For correspondence:

spdineshkumar@ucdavis.edu (SPD-K)

sma@ustc.edu.cn (SM)

## Supplementary Methods

### Gene co-expression network construction

Publically available human microarray datasets deposited in the ArrayExpress database [1] were used for network construction. Raw data (.cel) files from 986 datasets generated with the Affymetrix U133 plus 2.0 arrays were downloaded from the ArrayExpress database. The quality of these arrays were checked using arrayQualityMetrics in Bioconductor (http://www.bioconductor.org/) based on 6 scoring metrics: maplot, spatial, boxplot, heatmap, nuse, and rle [2]. After removing any arrays that failed two or more metrics, 9,947 high quality arrays from 948 datasets were kept for further analysis. The raw data of these arrays were then processed and normalized using the frozen robust multiarray analysis (fRMA) and combined into a single large expression matrix [3]. Probesets were annotated according to NCBI GEO database (GPL570), and 19,718 probesets representing 19,718 unique genes were selected for network analysis (Supplementary Dataset 8). Duplicated probesets for the same genes were removed because the presence of multiple probesets for the same genes will confound the partial correlation analysis.

The expression matrix containing the expression values of these 19,718 probesets/genes in 9,947 arrays were then used to construct a human gene co-expression network based on the graphical Gaussian model as described in [4]. Briefly, a shrinkage approach implemented in the GeneNet package in R was used to infer partial correlation between genes [5]. Because GeneNet only works with a limited number of genes at a time, a random sampling strategy was used, in which 2,000 genes were randomly selected and subjected to *pcor* analysis via GeneNet. After 3,000 rounds of random sampling, every

gene pair appears in more than a dozen sampling rounds with different calculated *pcors*, and the one with the lowest absolute value is chosen as the final *pcor* for that gene pair. The chosen *pcor* represents the *pcor* after removing the maximum amount of effect from other genes. The false discover rate (FDR) for the *pcor* analysis were calculated using fdrtool [6]. The resulted 186,132 significantly correlated gene pairs between 19,312 genes, with *pcor* >= 0.04, were chosen for gene co-expression network construction (Supplementary Dataset 9).

**Promoter motif analysis and target gene identification**

The transcription starting site (TSS) for the 19,718 analyzed genes were obtained from UCSC genome database or NCBI RefGene database. Promoter sequences are then extracted as 2500 bp upstream of TSS and 200 bp downstream of TSS, as listed in Supplementary Dataset 10, except for the analysis of NF-Y motif. For NF-Y motif, due to its high prevalence, promoter sequences are defined as 1000 bp upstream of TSS. For all promoter sequences, the repeat sequences and transposon sequences were replaced with "N" and excluded from motif analysis, as defined by the RepeatMasker or simple repeat tracks from UCSC.

Promoter motif analysis was carried out over the co-expression network to identify target genes for a specific motif. For every gene with >5 connected genes, the gene itself and its neighbor gene are considered as a group. Promoter motif position weight matrix (PWM) models are obtained from JASPAR, or from those motifs derived from the ENCODE project [7,8]. For the motifs derived from ENCODE project, only those corresponding to the known motifs were used. The promoters were then scanned for the

presence of the motifs using the POSSUM program, with a pValue cut off chosen at 4^-8 [9,10]. Motif enrichment and motif position bias analysis were then conducted for the group's promoters. If the motif is enriched or has significant biased distribution towards TSS with these promoters and the seeded gene contains the motif, all the genes containing the motifs within the group are considered to be targets genes of the motif.

Motif enrichment was analyzed based on the hypergeometric distribution. Suppose the analyzed promoters group contains *M* promoters in total, and a motif presents in *m* promoters among them. And in the *K* promoters for all the genes used in the human network analysis, the motif presents in *k* promoters. A pValue for that motif and gene group combination is calculated as:

$$pValue = \sum_{l=m}^{\min(k,M)} \frac{\binom{k}{l}\binom{K-k}{M-l}}{\binom{K}{M}} \qquad \text{Equation 5}$$

A pValue smaller or equal to selected cutoff value indicates the motif is enriched within the group.

Motif position bias towards TSS was assessed on the extent the motif's distribution deviated from random uniform distribution. For this purpose, a background model for a uniformly distributed motif in every gene promoter group was first established to calculate that motif's expected distance from TSS and its variance. Suppose the promoters are defined as *M* bp upstream of TSS, and *N* bp downstream of TSS. In all promoters in the group, there are *K* base pairs in total that are not occupied by repeat or transposon sequences. The motif has equal chance to appear in any of these *K* base pairs. Suppose in position $i$ ($-M <= i <= N$) along the promoters, which is $i$ bp relative to TSS, there are $k_i$ promoters is not occupied by repeat or transposon sequences in this position. Then, if the

motif only appears once in the promoter group, its expected mean distance from TSS is given by:

$$E(d) = \sum_{i=-M}^{N} \frac{k_i}{K} \times |i| \qquad \text{Equation 6}$$

And its variance is given by:

$$V(d) = \sum_{i=-M}^{N} \frac{k_i}{K} \times i^2 - (E(d))^2 \qquad \text{Equation 7}$$

*E(d)* and *V(d)* are calculated for every gene group in the network. For an actual motif appears *n* times in a group of promoters, with mean distance $\overline{|x|}$ from the TSS, a Z score is calculated as:

$$Z = \frac{E(d) - \overline{|x|}}{\sqrt{\frac{V(d)}{n}}} \qquad \text{Equation 8}$$

A Z score larger or equal to selected cutoff value is considered to have significant position bias towards TSS.

To assess the FDR in the analysis, the promoter sequences were permutated and the same analysis was conducted. After 100 rounds of permutation and analysis, the result is summarized to obtain the average identified target numbers for FDR rate calculation.

All the calculations were carried out with in-house developed scripts.

**Co-expression modules GO, TF binding, H3K4me3, methylation, and RNA-Seq data analyses**

The target genes for a specific motif were used to extract a sub-network from the entire human gene co-expression network. The sub-network was clustered using the MCL

clustering algorithm and visualized using BioLayout Express 3D [11,12]. Gene ontology analysis was also carried out using the GOStats package in Bioconductor [13].

To evaluate the enrichment of corresponding TFs binding to its motifs within the promoters of co-expression modules, the ENCODE TF-binding data were used [14]. The TF ChIP-Seq Uniform Peaks from ENCODE were downloaded (http://hgdownload.cse.ucsc.edu/goldenPath/hg19/encodeDCC/wgEncodeAwgTfbsUniform/). The binding region for a TF within a promoter was checked to see if it contains the corresponding motif sites. Only those promoters containing the TF's bound motif sites were considered to have a direct interaction with the corresponding TF. A hypergeometric test was then used to evaluate if a gene co-expression module have enriched direct binding to the TF compared to all the genes used for network analysis.

The H3K4me3 data were also downloaded from the ENCODE project (ENCODE UW Histone data, http://genome.ucsc.edu/cgi-bin/hgTrackUi?db=hg19&g=wgEncodeUwHistone). For every gene in each cell line, the peaks values between -2500 bp upstream of TSS to 200 bp downstream of TSS were summarized as the gene's H3K4me3 level in that cell type. The RNA-Seq data were also downloaded from ENCODE, and the RFKM values were used as the expression level of the gene. Similarly, promoter DNA methylation data were also downloaded from ENCODE. The CpG methylation data by methyl 450K beads arrays from HAIB were used (ENCODE HAIB Methyl450 data, http://genome.ucsc.edu/cgi-bin/hgTrackUi?db=hg19&g=wgEncodeHaibMethyl450). For a specific promoter in a specific tissue, the methylation levels for the residues within -500 bp to TSS were collected, and their 3rd quartile was calculated as the methylation level for that promoter, using the

BEDOPS software [15]. The RNA-Seq data were also downloaded from the ENCODE project (ENCODE Caltech RNA-Seq data, http://hgdownload.cse.ucsc.edu/goldenPath/hg19/encodeDCC/wgEncodeCaltechRnaSeq/ ).

## References


1 Rustici, G. *et al.* ArrayExpress update--trends in database growth and links to data analysis tools. *Nucleic acids research* **41**, D987-990, doi:10.1093/nar/gks1174 (2013).

2 Kauffmann, A., Gentleman, R. & Huber, W. arrayQualityMetrics--a bioconductor package for quality assessment of microarray data. *Bioinformatics* **25**, 415-416, doi:10.1093/bioinformatics/btn647 (2009).

3 McCall, M. N., Bolstad, B. M. & Irizarry, R. A. Frozen robust multiarray analysis (fRMA). *Biostatistics* **11**, 242-253, doi:10.1093/biostatistics/kxp059 (2010).

4 Ma, S., Gong, Q. & Bohnert, H. J. An Arabidopsis gene network based on the graphical Gaussian model. *Genome research* **17**, 1614-1625, doi:10.1101/gr.6911207 (2007).

5 Schäfer, J. & Strimmer, K. A shrinkage approach to large-scale covariance matrix estimation and implications for functional genomics. *Stat Appl Genet Mol Biol* **4**, Article32, doi:10.2202/1544-6115.1175 [doi] (2005).

6 Strimmer, K. fdrtool: a versatile R package for estimating local and tail area-based false discovery rates. *Bioinformatics* **24**, 1461-1462, doi:10.1093/bioinformatics/btn209 (2008).

7 Kheradpour, P. & Kellis, M. Systematic discovery and characterization of regulatory motifs in ENCODE TF binding experiments. *Nucleic acids research* **42**, 2976-2987, doi:10.1093/nar/gkt1249 (2014).

8 Mathelier, A. *et al.* JASPAR 2014: an extensively expanded and updated open-access database of transcription factor binding profiles. *Nucleic acids research* **42**, D142-147, doi:10.1093/nar/gkt997 (2014).

9 Touzet, H. & Varre, J. S. Efficient and accurate P-value computation for Position Weight Matrices. *Algorithms for molecular biology : AMB* **2**, 15, doi:10.1186/1748-7188-2-15 (2007).

10 Fu, Y., Frith, M. C., Haverty, P. M. & Weng, Z. MotifViz: an analysis and visualization tool for motif discovery. *Nucleic acids research* **32**, W420-423, doi:10.1093/nar/gkh426 (2004).

11 Enright, A. J., Van Dongen, S. & Ouzounis, C. A. An efficient algorithm for large-scale detection of protein families. *Nucleic acids research* **30**, 1575-1584 (2002).

12 Theocharidis, A., van Dongen, S., Enright, A. J. & Freeman, T. C. Network visualization and analysis of gene expression data using BioLayout Express(3D). *Nat Protoc* **4**, 1535-1550, doi:10.1038/nprot.2009.177 (2009).

13 Falcon, S. & Gentleman, R. Using GOstats to test gene lists for GO term association. *Bioinformatics* **23**, 257-258, doi:10.1093/bioinformatics/btl567 (2007).

14 Encode Project Consortium *et al.* An integrated encyclopedia of DNA elements in the human genome. *Nature* **489**, 57-74, doi:10.1038/nature11247 (2012).

15 Neph, S. *et al.* BEDOPS: high-performance genomic feature operations. *Bioinformatics* **28**, 1919-1920, doi:10.1093/bioinformatics/bts277 (2012).

**Titles and legends of supplementary figures and datasets**

**Supplementary Figure S1. NF-YA's depletion up-regulated or down-regulated selected NF-YA motif modules in human cell lines.**

Two published microarray datasets (GSE40215 and GSE70543) were analyzed using GSEA v2.2.0. **a)** GSEA enrichment plot showing that NF-Y motif module #1 was down-regulated in NF-YA depleted HeLa S3 cells. **b)** GSEA enrichment plot showing that NF-Y motif module #10 was up-regulated in NF-YA depleted HeLa S3 cells. **c)** The list of NF-Y motif modules that were up- or down-regulated in NF-YA depleted HeLa S3 cells. **d)** The list of NF-Y motif modules that were down-regulated in NF-YA depleted HCT116 cells.

**Supplementary Figure S2. The gene expression patterns of Sp1 motif-regulated modules in various human primary cell lines.**

The gene expression heatmap of Sp1 motif-regulated modules was generated using the expression data obtained from GSE49910, which includes the gene expression profiles of 116 human primary cell lines using Affymetrix Human Genome U133 Plus 2.0 microarray slides [8]. Rows represent genes and columns represent cell lines. The color scale shown at top left indicates gene expression levels. Genes are grouped by modules.

**Supplementary Figure S3. Promoter H3K4me3 and DNA methylation levels in Sp1 motif-regulated modules.**

**a)** The average H3K4me3 levels in each module among 59 different cell lines as measured by the ENCODE project.

**b)** The average DNA methylation level in each module among 63 different cell lines as measured by the ENCODE project.

**c)** The median H3K4me3 levels for all genes in module #85 in each of the 59 cell lines, sorted from highest to lowest.

**d)** The average DNA methylation levels for all genes in module #85 in each of the 63 cell lines, sorted from highest to lowest.

**e)** The median H3K4me3 levels for all genes in module #5 in each of the 59 cell lines, sorted from highest to lowest.
**f)** The average DNA methylation levels for all genes in module #5 in each of the 63 cell lines, sorted from highest to lowest.

**Supplementary Dataset 1: Modules identified from the whole co-expression human network**

**Supplementary Dataset 2: Immunity-related modules identified from the whole co-expression network**

**Supplementary Dataset 3: NF-Y motif-regulated modules**

**Supplementary Dataset 4: The median gene expression levels of the NF-Y modules in three human cell lines**

**Supplementary Dataset 5: Sp1 motif-regulated modules**

**Supplementary Dataset 6: Selected genes from the Sp1 development modules that have been reported to be regulated by the Sp1 motif**

**Supplementary Dataset 7: Target gene list for all the analyzed motifs**

**Supplementary Dataset 8: Genes used in the network construction**

**Supplementary Dataset 9: The gene pairs used in the GGM gene co-expression network construction**

**Supplementary Dataset 10: The promoter regions of the genes used in the analysis**

## Supplementary Figures

**Supplementary Figure S1. NF-YA's depletion up-regulated or down-regulated selected NF-YA motif modules in human cell lines.**

Two published microarray datasets (GSE40215 and GSE70543) were analyzed using GSEA v2.2.0. **a)** GSEA enrichment plot showing that NF-Y motif module #1 was down-regulated in NF-YA depleted HeLa S3 cells. **b)** GSEA enrichment plot showing that NF-Y motif module #10 was up-regulated in NF-YA depleted HeLa S3 cells. **c)** The list of NF-Y motif modules that were up- or down-regulated in NF-YA depleted HeLa S3 cells. **d)** The list of NF-Y motif modules that were down-regulated in NF-YA depleted HCT116 cells.

(See Supplementary Figure S1 on next page)

**a**

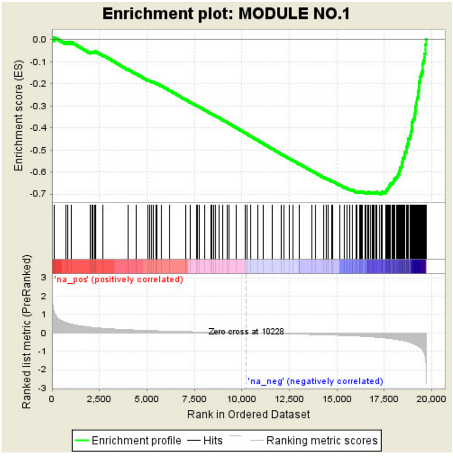


**b**

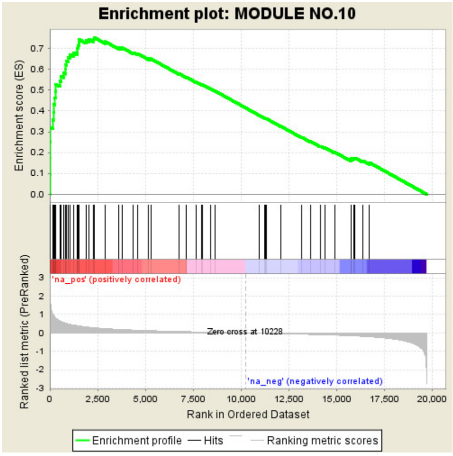


**c**

NF-YA depleted HeLa S3 cells vs. Control

| Module No. | SIZE | ES | NES | NOM p-value | FDR q-value |
|---|---|---|---|---|---|
| Up-regulated modules | | | | | |
| 10 | 58 | 0.75 | 2.50 | 0 | 0 |
| 55 | 18 | 0.89 | 2.29 | 0 | 0 |
| 42 | 22 | 0.83 | 2.22 | 0 | 0 |
| Down-regulated modules | | | | | |
| 1 | 180 | -0.70 | -2.80 | 0 | 0 |
| 8 | 64 | -0.64 | -2.18 | 0 | 0 |
| 37 | 25 | -0.68 | -1.94 | 0 | 0.005825991 |

**d**

NF-YA depleted HCT116 cells vs. Control

| Module No. | SIZE | ES | NES | NOM p-value | FDR q-value |
|---|---|---|---|---|---|
| Down-regulated modules | | | | | |
| 7 | 65 | -0.68 | -2.32 | 0 | 0 |
| 11 | 54 | -0.58 | -1.93 | 0 | 0.009275232 |
| 18 | 44 | -0.58 | -1.90 | 0 | 0.01308954 |
| 34 | 26 | -0.63 | -1.83 | 0.001477105 | 0.030749764 |
| 47 | 21 | -0.65 | -1.82 | 0.007473842 | 0.026191939 |
| 49 | 21 | -0.65 | -1.82 | 0.004424779 | 0.023488693 |

# Figure S1

**Supplementary Figure S2. The gene expression patterns of Sp1 motif-regulated modules in various human primary cell lines**

The gene expression heatmap of Sp1 motif-regulated modules was generated using the expression data obtained from GSE49910, which includes the gene expression profiles of 116 human primary cell lines using Affymetrix Human Genome U133 Plus 2.0 microarray slides [8]. Rows represent genes and columns represent cell lines. The color scale shown at top left indicates gene expression levels. Genes are grouped by modules.

(See Supplementary Figure S2 on next page)

# Sp1 Motif−Regulated Modules

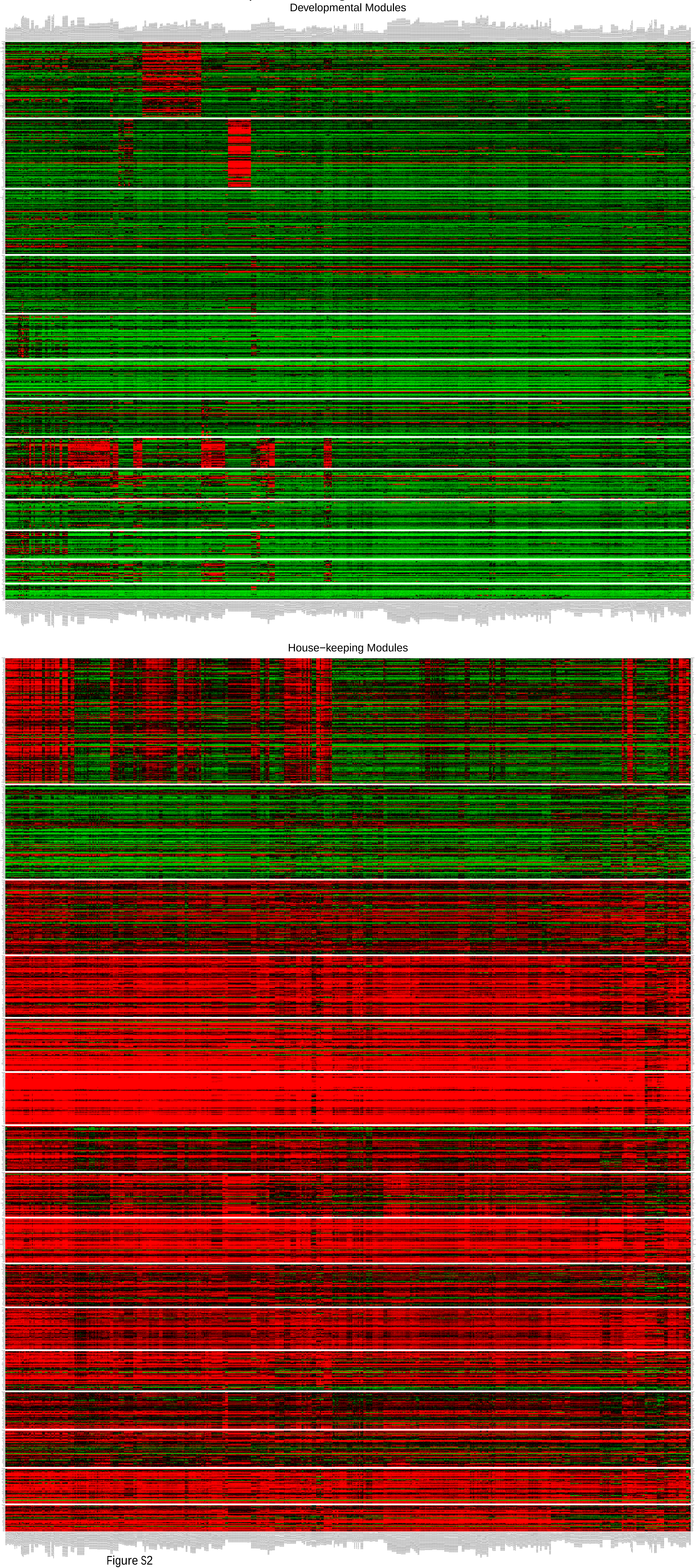


Figure S2

**Supplementary Figure S3. Promoter H3K4me3 and DNA methylation levels in Sp1 motif-regulated modules.**

**a)** The average H3K4me3 levels in each module among 59 different cell lines as measured by the ENCODE project.

**b)** The average DNA methylation level in each module among 63 different cell lines as measured by the ENCODE project.

**c)** The median H3K4me3 levels for all genes in module #85 in each of the 59 cell lines, sorted from highest to lowest.

**d)** The average DNA methylation levels for all genes in module #85 in each of the 63 cell lines, sorted from highest to lowest.

**e)** The median H3K4me3 levels for all genes in module #5 in each of the 59 cell lines, sorted from highest to lowest.

**f)** The average DNA methylation levels for all genes in module #5 in each of the 63 cell lines, sorted from highest to lowest.

(See Supplementary Figure S3 on next gage)

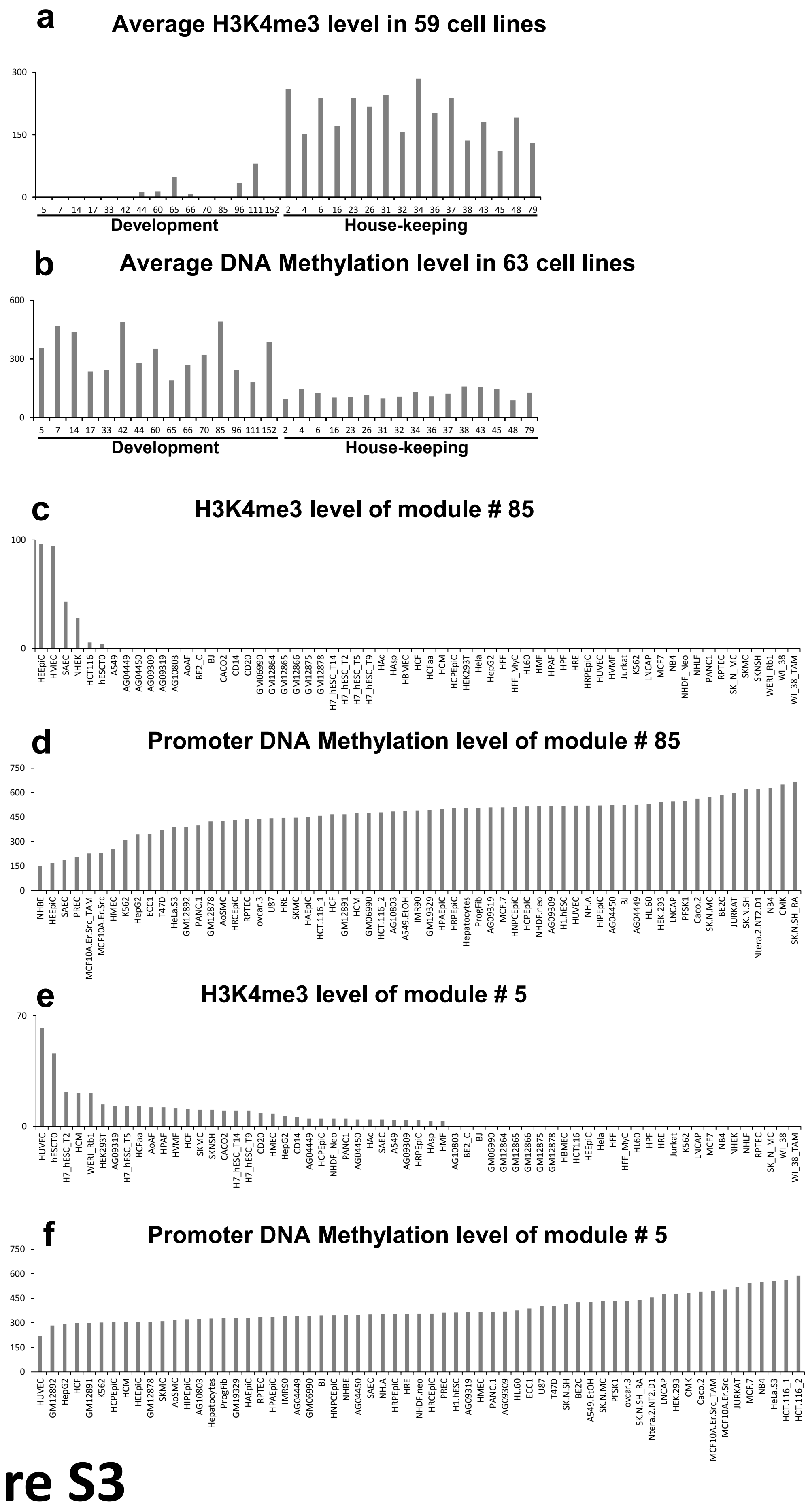
a
Average H3K4me3 level in 59 cell lines
300
150
0
5 7 14 17 33 42 44 60 65 66 70 85 96 111 152 2 4 6 16 23 26 31 32 34 36 37 38 43 45 48 79
Development
House-keeping
b
Average DNA Methylation level in 63 cell lines
600
300
0
5 7 14 17 33 42 44 60 65 66 70 85 96 111 152 2 4 6 16 23 26 31 32 34 36 37 38 43 45 48 79
Development
House-keeping
c
H3K4me3 level of module # 85
100
0
HEEpiC HMEC SAEC NHEK HCT116 hESCT0 A549 AG04449 AG04450 AG09309 AG09319 AG10803 AoAF BE2_C BJ CACO2 CD14 CD20 GM06990 GM12864 GM12865 GM12866 GM12875 GM12878 H7_hESC_T14 H7_hESC_T2 H7_hESC_T5 H7_hESC_T9 HAc HAsp HBMEC HCF HCFaa HCM HCPEpiC HEK293T Hela HepG2 HFF HFF_MyC HL60 HMF HPAF HPF HRE HRPEpiC HUVEC HVMF Jurkat K562 LNCAP MCF7 NB4 NHDF_Neo NHLF PANC1 RPTEC SK_N_MC SKMC SKNSH WERI_Rb1 WI_38 WI_38_TAM
d
Promoter DNA Methylation level of module # 85
750
600
450
300
150
0
NHBE HEEpiC SAEC PREC MCF10A.Er.Src_TAM MCF10A.Er.Src HMEC K562 HepG2 ECC1 T47D HeLa.S3 GM12892 PANC.1 GM12878 AoSMC HRCEpiC RPTEC ovcar.3 U87 HRE SKMC HAEpiC HCT.116_1 HCF GM12891 HCM GM06990 HCT.116_2 AG10803 A549.EtOH IMR90 GM19329 HPAEpiC HRPEpiC Hepatocytes ProgFib AG09319 MCF.7 HNPCEpiC HCPEpiC NHDF.neo AG09309 H1.hESC HUVEC NH.A HIPEpiC AG04450 BJ AG04449 HL.60 HEK.293 LNCAP PFSK1 Caco.2 SK.N.MC BE2C JURKAT SK.N.SH Ntera.2.NT2.D1 NB4 CMK SK.N.SH_RA
e
H3K4me3 level of module # 5
70
0
HUVEC hESCT0 H7_hESC_T2 HCM WERI_Rb1 HEK293T AG09319 H7_hESC_T5 HCFaa AoAF HPAF HVMF HCF SKMC SKNSH CACO2 H7_hESC_T14 H7_hESC_T9 CD20 HMEC HepG2 CD14 AG04449 HCPEpiC NHDF_Neo PANC1 AG04450 HAc SAEC A549 AG09309 HRPEpiC HAsp HMF AG10803 BE2_C BJ GM06990 GM12864 GM12865 GM12866 GM12875 GM12878 HBMEC HCT116 HEEpiC Hela HFF HFF_MyC HL60 HPF HRE Jurkat K562 LNCAP MCF7 NB4 NHEK NHLF RPTEC SK_N_MC WI_38 WI_38_TAM
f
Promoter DNA Methylation level of module # 5
750
600
450
300
150
0
HUVEC GM12892 HepG2 HCF GM12891 K562 HCPEpiC HCM HEEpiC GM12878 SKMC AoSMC HIPEpiC AG10803 Hepatocytes ProgFib GM19329 HAEpiC RPTEC HPAEpiC IMR90 AG04449 GM06990 BJ HNPCEpiC NHBE AG04450 SAEC NH.A HRPEpiC HRE NHDF.neo HRCEpiC PREC H1.hESC AG09319 HMEC PANC.1 AG09309 HL.60 ECC1 U87 T47D SK.N.SH BE2C A549.EtOH SK.N.MC PFSK1 ovcar.3 SK.N.SH_RA Ntera.2.NT2.D1 LNCAP HEK.293 CMK Caco.2 MCF10A.Er.Src_TAM MCF10A.Er.Src JURKAT MCF.7 NB4 HeLa.S3 HCT.116_1 HCT.116_2

# Figure S3
